# Identifying risk and response to nutritional supplementation in severely underweight children missed by standard treatment criteria in Mali: A secondary prognostic analysis of data from a randomised controlled trial

**DOI:** 10.1371/journal.pgph.0007525

**Published:** 2026-11-12

**Authors:** Isabel Potani, Abel Khisa, Zachary Tausanovitch, Grace Heymsfield, Christian Ritz, Debbie Thompson, Robert H. J. Bandsma, André Briend, Osborne Potani, Geoffrey Manda, Mphatso Chisala, Emily Bamusi-Nkhoma, Jeanette Bailey, Suvi T. Kangas

**Affiliations:** 1 Department of Global Health, Ripple Global Health Ltd, London, United Kingdom; 2 Airbel Impact Lab, International Rescue Committee, New York, New York, United States of America; 3 National Institute of Public Health, University of Southern Denmark, Copenhagen, Denmark; 4 Tropical Metabolism Research Unit, The University of the West Indies, Kingston, Jamaica; 5 Centre for Global Child Health, Hospital for Sick Children, Toronto, Canada; 6 Tampere Centre for Child, Adolescent, and Maternal Health Research, Faculty of Medicine and Health Technology, Tampere University and Tampere University Hospital, Tampere, Finland; 7 Department of Nutrition, Exercise, and Sports, Faculty of Science, University of Copenhagen, Copenhagen, Denmark; 8 Institute of Epidemiology and Health, University College London, London, United Kingdom; 9 Airbel Impact Lab, International Rescue Committee, Brussels, Belgium; Xi’an Jiaotong University, CHINA

## Abstract

Severely underweight children (weight-for-age Z-score [WAZ] <-3) are ineligible for wasting treatment unless they have a mid-upper arm circumference (MUAC) <125 mm or a weight-for-height Z-score < -3. However, the risk of negative outcomes without nutritional supplementation and the likelihood of positive outcomes with supplementation are unknown. We aimed to investigate this using a secondary analysis of randomised controlled trial data from children in Mali with WAZ < -3 and MUAC ≥ 125 mm. We assessed baseline anthropometric, clinical, and social factors associated with negative outcomes (sustained WAZ change < 0 at any point during 0–12 weeks of follow-up or death or hospitalisation, or development of MUAC < 125 mm) in the absence of nutritional supplementation, and positive outcomes (sustained WAZ change ≥ 0.15 and no death and no hospitalisation, and sustained MUAC ≥ 125 mm) with nutritional supplementation. Unadjusted and adjusted generalised linear models were fitted separately for the supplemented and unsupplemented children, with significant exposures from the unadjusted models then adjusted for sex, season, age, and distance from the health centre. A total of 1378 children were included. Less than 2% of children experienced hospitalisation or death; thus, the negative outcomes were mostly anthropometric. Among children who were not nutritionally supplemented, 35% (151/432) experienced negative anthropometric outcomes, while 67% (643/946) of children who were supplemented experienced positive anthropometric outcomes. In the unadjusted analysis only, younger age (< 2 years), lower body weight and height, and currently breastfeeding children were associated with an increase in negative anthropometric outcomes. Among those receiving nutritional support, having an illness prior to the start of supplementation was associated with positive anthropometric outcomes in both unadjusted and adjusted analyses. Different factors were associated with negative and positive anthropometric outcomes, with no overlap, highlighting the difficulty of targeting nutritional supplementation among severely underweight children.

## Introduction

Undernutrition, e.g., being underweight, is associated with poor health outcomes such as growth faltering, delayed development, morbidity, and mortality in children under five years of age [1,2]. Undernutrition affects millions of children worldwide, particularly in low- and middle-income settings [1]. Underweight is defined as a weight-for-age Z-score (WAZ) <-2 standard deviations (SDs) from the median of the World Health Organisation (WHO) growth standards. Unlike severe wasting (diagnosed by weight-for-height Z-score (WHZ) <-3 and/or mid-upper arm circumference (MUAC) < 115 mm), severe underweight (WAZ < -3) alone is not an independent criterion for receiving nutritional supplementation, such as specially formulated food (SFF) [3]. However, both wasting and underweight are highly prevalent, affecting approximately 45 and 80 million children, respectively [4–6]. Furthermore, both severe underweight and severe wasting are associated with high mortality, 9 and 11 times higher than that of non-undernourished children, respectively [2].

While all severely undernourished children are at high risk of poor health outcomes [7], it is increasingly recognised that risk may not be uniform. Some severely undernourished children may have a greater risk of morbidity, mortality, and further or sustained undernutrition [8,9]. However, there is limited research on which severely undernourished children are more likely to deteriorate in the absence of treatment, and whether these children experience more favourable outcomes with nutritional supplementation. Given the high mortality risk among severely underweight children [7], use of WAZ < -3 as a first-line detection of children at risk of poor health outcomes has been suggested [8]. Severely underweight children with MUAC ≥125 mm represent an important subgroup that is likely to be missed by wasting treatment programmes, particularly those using MUAC-only admission criteria, i.e., MUAC < 125 mm. To further enhance the efficiency of delivering health interventions to this population, it is essential to identify children with WAZ < -3 who are at greatest risk of adverse outcomes without supplementation, and those who will experience positive health outcomes with supplementation.

The objectives of this study were to identify, among children with WAZ < -3 SD (severely underweight) and MUAC ≥ 125 mm:

1. Baseline characteristics associated with the development of negative outcomes (MUAC < 125 mm, death, hospitalisation, WAZ change < 0.0) within twelve weeks of follow-up in the absence of nutritional supplementation.
2. Baseline characteristics associated with positive outcomes (sustained WAZ change ≥ 0.15, no death or hospitalisation, nor development of MUAC < 125 mm within twelve weeks of follow-up in the presence of nutritional supplementation).
3. Baseline characteristics associated with both negative (as identified in Objective 1) and positive outcomes (as defined in Objective 2).

## Methods

### Study design

This study was a secondary analysis of an individually randomised controlled trial (RCT) conducted in Mali, from September 2022 to March 2024 (S1 CONSORT Checklist). The parent study enrolled children aged 6–59 months across 11 participating health centres [10]. All children enrolled in the parent trial were eligible for inclusion and were regrouped according to whether they received nutritional supplementation rather than by randomised arm; the analysis therefore does not preserve the randomised comparison.

### Study participants

In the primary RCT, after eligibility screening (MUAC ≥ 125 mm, WAZ < -3, and age 6–59 months) and informed consent, participants were randomly allocated to one of three study arms. These included: i) a control arm, which received no SFF; ii) a simplified treatment arm, in which all children received one sachet (500 kcal) of ready-to-use therapeutic food (RUTF) daily until WAZ ≥ -3; and iii) a standard treatment arm, where children with a WHZ < -3 were administered RUTF at a dosage of 125–215 kcal per kilogram of body weight per day until WHZ ≥ -2, children with WHZ ≥ -3 to < -2 were administered 1 daily sachet of RUTF, and children with WHZ ≥ -2 received no SFF.

Follow-up assessments were scheduled fortnightly for the initial twelve weeks of the intervention period and monthly thereafter, continuing until 6 months post-enrolment. At each follow-up visit, anthropometric measurements (weight, height, MUAC) and morbidity data were systematically collected. Furthermore, body composition, assessed via bioelectrical impedance analysis (BIA) and skinfold thickness measurements, and haemoglobin concentrations were measured at baseline and at 2- and 6-months post-enrolment. Participants assigned to the two intervention arms (simplified and standard) received treatment for up to 12 weeks. Treatment was discontinued earlier if a child achieved recovery (defined as WHZ ≥ -2 in the standard arm and WAZ ≥ -3 in the simplified arm, sustained for two consecutive visits), defaulted from the treatment (characterised by two missed successive treatment visits), necessitated inpatient care due to medical complications, developed a MUAC < 125 mm or oedema, or died. Individuals who developed a MUAC < 125 mm or oedema were referred for routine treatment according to the simplified protocol and were subsequently exited from the study [11].

In order to be programmatically relevant, this parent study used different recovery criteria for the simplified and standard treatment arms. In the simplified arm, recovery was declared with WAZ ≥ -3 upon two consecutive visits which was considered a programmatically logical criterion if WAZ < -3 was to be included as an admission criterion, and has also been used by others [12]. The standard treatment arm used the standard recovery definitions proposed by WHO for wasting treatment, meaning WHZ ≥ -2 [13].

No routine medications were provided as part of this study; medications were given only when clinically indicated and administered through routine clinic services rather than as part of the study intervention.

### Eligibility criteria

For this secondary analysis, all children in the main study were included, except those who withdrew consent. The included children were disaggregated into two subgroups based on whether they received any form of nutritional supplementation, i.e., the supplemented group and the unsupplemented group, as part of the secondary analysis (post hoc). Thus, allocation to supplemented and unsupplemented groups in this secondary analysis reflects actual receipt of supplementation rather than randomised allocation. Receipt of supplementation was determined jointly by the parent-trial arm, baseline WHZ category, and the trial’s recovery and treatment-discontinuation rules.

### Study setting

The study was conducted in the Nara health district of the Koulikoro region in southern Mali [10]. The district has a surface area of approximately 30,000 km² and an estimated population of 350,000 in 2021, with approximately 20% under 5 years of age [14]. The region's climate is dry, with livelihoods centred on smallholder agriculture and pastoralism. Compared with northern and central Mali, it has experienced lower levels of insecurity and conflict. Since 2018, food security has remained relatively stable, with food insecurity classified as minimal [15]. The 2023 national nutrition survey estimated that 3.9% of all 6–59-month-old children had MUAC < 125 mm and 4.1% had WAZ < -3 in the Koulikoro Region [16]. Within the same report, under-five mortality was reported to be approximately 0.11 deaths per 10,000 persons per day [16]. Exclusive breastfeeding among infants aged 0–6 months was reported at approximately 70%, and the proportion reaching minimum dietary diversity among infants aged 6–23 months was approximately 21% [16].

The predominant childhood illnesses in Mali are malaria, acute respiratory infection and diarrhoeal disease [17–19]. Malaria is highly seasonal in this Sahelian zone and is a leading cause of childhood morbidity and mortality in Mali, accounting for approximately 37% of outpatient consultations and 33% of health-facility deaths in 2021; children aged 3–59 months in the region receive seasonal malaria chemoprevention between June and October [17,18]. In the most recent national survey data, the two-week prevalence among children under five in Mali was approximately 13% for symptoms of acute respiratory infection and 17% for diarrhoea [17,20].

### Data extraction and preparation

Using R software version 4.5.1 [21], we extracted and prepared demographic, anthropometric, biochemical, clinical, body composition, household, and geographical data collected from enrolment through the study's conclusion. Key variables were selected for analysis, and data quality and consistency were checked prior to modelling. Detailed data-processing procedures are provided in S1 Table.

### Outcomes

The negative and positive outcomes were assessed every two weeks from enrolment to 12 weeks after enrolment (the maximum duration of supplementation). The assessment endpoints and time points also considered negative/positive outcomes occurring within ±2 weeks of each time point to account for missed visits at the exact follow-up time point.

#### Negative outcomes in the unsupplemented group.

To define the negative outcome, we used a composite binary variable (yes/no). A negative outcome (yes) was defined as the occurrence of any of the following at any point during the follow-up period: death, severe illness (defined as any of severe acute respiratory infection, complicated malaria, diarrhoea with dehydration), development of oedema or MUAC < 125 mm, or deterioration of WAZ (WAZ change < 0.0) at any point during the 0–12 weeks of follow-up in the unsupplemented group. Because death and severe illness were uncommon in this cohort, this composite outcome was driven predominantly by anthropometric change and is therefore referred to as a negative anthropometric outcome. The WAZ-change threshold used to define it was pragmatic rather than clinically validated.

#### Positive outcomes in the supplemented group.

To define the positive outcome, we used a composite binary variable (yes/no). A positive outcome (yes) was defined as a sustained improvement in weight-for-age (WAZ change ≥ 0.15) together with the absence of all of the following during the 0–12 weeks of follow-up: death, severe illness (defined as any of severe acute respiratory infection, complicated malaria, or diarrhoea with dehydration), and development of oedema or MUAC < 125 mm, in the supplemented group. As death and severe illness were uncommon, this outcome was driven predominantly by anthropometric change and is therefore referred to as a positive anthropometric outcome.

### Exposures of interest

The full list of features considered for modelling as exposures for negative and positive anthropometric outcomes included child-level, caregiver, and household factors, all measured at enrolment (S1 Table). Child-level factors included age, haemoglobin concentration, illnesses, anthropometry (WHZ, MUAC, height-for-age Z-score [HAZ]), body composition (fat-free mass, fat mass, fat-free mass index, fat mass index, triceps skinfold z-score, sub-scapular skinfold Z-score), and diet (breastfeeding, dietary diversity, and hunger) at enrolment. Caregiver factors included education. Household factors included household hunger and receipt of food aid. Household hunger was assessed using the Household Hunger Scale (HHS) methodology by the Food and Nutrition Technical Assistance (FANTA) Project [22].

### Data analysis

Categorical variables were reported as counts and percentages. For continuous data, descriptive statistics were reported as median and interquartile range (IQR). We estimated unadjusted and adjusted risk ratios using generalised linear models with a Poisson distribution and a log link, separately for supplemented and unsupplemented groups. Robust standard errors were used to account for variance misspecification due to the binary outcome. Statistical significance for all tests was set at p < 0.05, and 95% confidence intervals were reported for all regression model estimates. For the adjusted analysis, we assessed all candidate exposure variables with p < 0.05 in the unadjusted analysis. The adjusted models included the child's age, sex, season, and distance to the health facility as covariates in all models, and the dosage of nutritional supplementation was additionally included in models restricted to children who received nutritional supplementation. The estimand for both models was a prognostic association within groups defined by exposure, rather than a treatment effect. For each exposure-defined group (unsupplemented and supplemented), we estimated the association between each baseline characteristic measured at enrolment and the probability of the group-specific composite outcome over the 0–12-week follow-up. Among unsupplemented children, the outcome was the negative anthropometric outcome; among supplemented children, it was the positive anthropometric outcome summarised as a risk ratio. We screened candidate exposures by univariable significance before adjustment and examined many exposures, so the analysis is exploratory and hypothesis-generating. The supplemented and unsupplemented groups are not exchangeable, and no causal comparison was made between them. Each model was fitted using complete-case data for the relevant exposure, and the number of children contributing to each model is reported in the corresponding table. Outcome status was assigned from each child’s last available anthropometric measurement (last observation carried forward), so children not completing 12 weeks contributed their most recent visit. Children referred for routine treatment on developing MUAC < 125 mm or oedema, requiring inpatient care for medical complications, or who died were classified as having a negative anthropometric outcome irrespective of their weight-for-age trajectory.

### Sensitivity analyses

Sensitivity analyses were conducted. Given the absence of an established minimum clinically meaningful change in WAZ, we varied the thresholds used to define positive (≥ 0.10, ≥ 0.20) and negative (< -0.10, < -0.05) outcomes relative to the primary thresholds of ΔWAZ ≥ 0.15 (positive) and < 0 (negative). We additionally excluded children lost to follow-up, who recovered, or who defaulted, compared outcome definitions that included versus excluded morbidity and mortality, and restricted analyses to anthropometric outcomes only. We also explored alternative model specifications using Akaike Information Criterion (AIC)-based model selection, refitting each adjusted model with bidirectional stepwise AIC selection (k = 2), bounded by the intercept-only and full models, with the exposure eligible for removal. In a post-hoc, non-pre-specified descriptive analysis, we tabulated the negative anthropometric outcome by age band at enrolment among children in the unsupplemented group who were currently breastfeeding at enrolment, to explore whether such outcomes occurred predominantly at ages when complementary foods are typically introduced; no formal test of association was performed, and exclusive breastfeeding duration and age at introduction of complementary foods were not recorded.

## Results

A total of 1397 children aged 6–59 months with WAZ < -3 SD and MUAC ≥ 125 mm were recruited in the primary study (Fig 1). In both the primary analysis and this secondary analysis, 19 children were excluded for ineligibility because they had a WAZ ≥ -3 SD at enrolment. Thus, a total of 1,378 children were included in the secondary analysis, comprising 946 who received nutritional supplementation and 432 who did not.

**Fig 1:**
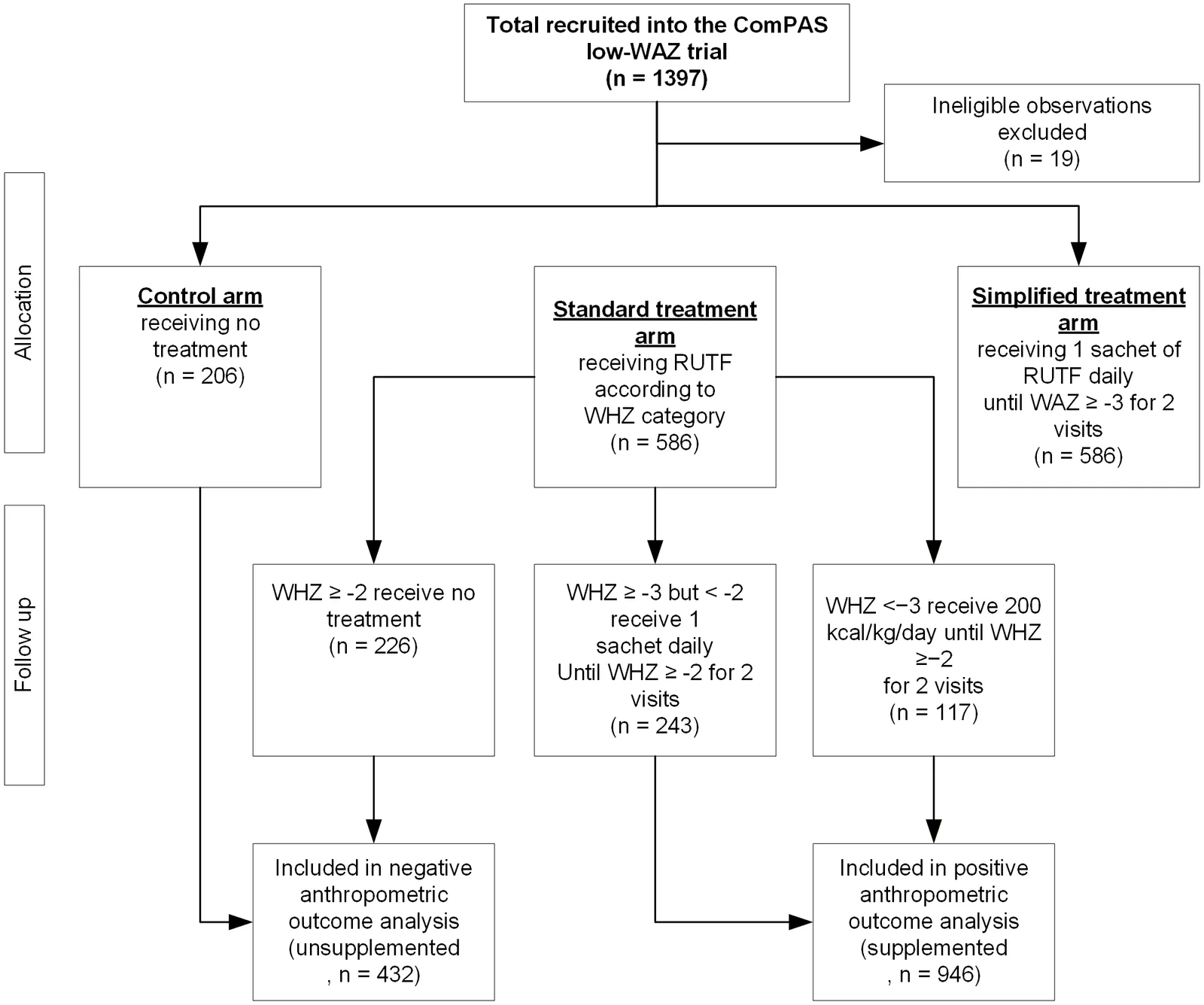
Inclusion of participants in the secondary analysis. Abbreviations: MUAC, mid-upper arm circumference; RUTF, ready-to-use therapeutic food; SFF, specially formulated food; WAZ, weight-for-age Z-score; WHZ, weight-for-height Z-score.

The median [Q1, Q3] age of children at enrolment was 31 [24, 40] months, and 57% were boys (Table 1). The median [Q1, Q3] WHZ was -2.1 [-2.6, -1.6]; the median [Q1, Q3] HAZ was -3.5 [-4.1, -3.0], and the median [Q1, Q3] MUAC was 128.5 mm [126, 131.5] at enrolment. Additional baseline characteristics are presented in S2 Table.

**Table 1:** Baseline characteristics among severely underweight children.

	Unsupplemented group	Supplemented group	All children	
Characteristic at Enrolment	Median[IQR]/n(%)	Median[IQR]/n(%)	Median[IQR]/n(%)	N(%)
Total (n) %	432 (100%)	946 (100%)	1378 (100%)	
**Demographic**				
Age (months)	33 [25, 41]	30 [23, 40]	31 [24, 40]	1378 (100.0%)
< 24 months old	327 (76%)	250 (26%)	577 (42%)	1378 (100.0%)
Male	212 (49%)	572 (60%)	784 (57%)	1378 (100.0%)
**Anthropometry and Body Composition**				
Weight (kg)	8.9 [8.0, 9.8]	8.6 [7.8, 9.7]	8.8 [7.8, 9.8]	1378 (100.0%)
Height (cm)	79.4 [75.0, 83.6]	79.5 [75.0, 85.2]	79.4 [75.0, 84.5]	1378 (100.0%)
WHZ	-1.7 [-2.0, -1.3]	-2.2 [-2.7, -1.9]	-2.1 [-2.6, -1.6]	1378 (100.0%)
Severe wasting (WHZ < -3 SD)	22 (5%)	98 (10%)	120 (9%)	1378 (100.0%)
WAZ	-3.3 [-3.6, -3.2]	-3.4 [-3.6, -3.2]	-3.4 [-3.6, -3.2]	1378 (100.0%)
HAZ	-3.8 [-4.3, -3.3]	-3.4 [-3.9, -2.9]	-3.5 [-4.1, -3.0]	1378 (100.0%)
Severe stunting (HAZ < -3 SD)	373 (86%)	652 (69%)	1025 (74%)	1378 (100.0%)
MUAC (mm)	129 [126.5, 132.5]	128 [126, 131]	128.5 [126, 131.5]	1378 (100.0%)
FFM (kg)†	(155) 6.6 [6.1, 7.3]	(294) 6.5 [6.0, 7.2]	6.5 [6.0, 7.2]	449 (32.6%)
FM (kg)†	(149) 2.2 [1.3, 2.8]	(288) 1.9 [1.3, 2.6]	2.0 [1.3, 2.6]	437 (31.7%)
Triceps skinfold thickness (Z-score)	-0.7 [-1.2, -0.1]	-0.8 [-1.4, -0.3]	-0.8 [-1.3, -0.2]	1378 (100.0%)
Subscapular skinfold thickness (Z-score)	-0.7 [-1.4, 0.0]	-0.9 [-1.7, -0.2]	-0.8 [-1.6, -0.1]	1378 (100.0%)
**Clinical**				
Haemoglobin (g/dL)†	(427) 8.7 [7.3, 10.1]	(937) 8.9 [7.5, 10.2]	8.8 [7.5, 10.2]	1364 (99.0%)
Has anaemia: [Hb < 11 g/dL]†	376/427 (88%)	824/937 (88%)	1200 (88%)	1364 (99.0%)
Illness at enrolment	168 (39%)	407 (43%)	575 (42%)	1378 (100.0%)
**Infant and Young Child Feeding**				
Currently breastfeeding	91 (21%)	236 (25%)	327 (24%)	1378 (100.0%)
Dietary Diversity Score	2 [1, 3]	2 [1, 3]	2 [1, 3]	1378 (100.0%)
Attained the Minimum Dietary Diversity Score	2 (0%)	10 (1%)	12 (1%)	1378 (100.0%)
Self-feeding	407 (94%)	843 (89%)	1250 (91%)	1378 (100.0%)
**Dosage of Nutritional Supplementation**				
No intervention	432 (100%)	--	432 (31%)	1378 (100.0%)
1 RUTF/day until WHZ > -2 SD	--	243 (26%)	243 (18%)	1378 (100.0%)
1 RUTF/day until WAZ ≥ -3 SD	--	586 (62%)	586 (43%)	1378 (100.0%)
125-215 kcal/kg/day until WHZ ≥ -2	--	117 (12%)	117 (8%)	1378 (100.0%)
**Caregiver and Household Characteristics**				
Household head employment – unemployed†	19/380 (5%)	38/825 (5%)	57 (5%)	1205 (87.4%)
Household size	13 [7,20]	13 [8, 22]	13 [8, 21]	1378 (100.0%)
HHS – Little to no hunger	402 (93%)	879 (93%)	1281 (93%)	1378 (100.0%)
Lean season	208 (48%)	494 (52%)	702 (51%)	1378 (100.0%)
Received food aid (past month or two weeks)	24 (6%)	54 (6%)	78 (6%)	1378 (100.0%)

Abbreviations: FFM, fat-free mass; FFMI, fat-free mass index; FM, fat mass; FMI, fat mass index; HAZ, height-for-age z-score; HHS, household hunger score; MUAC, mid-upper arm circumference; RUTF, ready-to-use therapeutic food; SES, socioeconomic status; WAZ, weight-for-age z-score; WHZ, weight-for-height z-score. Sample sizes are shown in parentheses only when there is missing data. A dash (--) indicates zero observations

In the unsupplemented group, 35% (151/432) of the children experienced negative anthropometric outcomes, whereas 67% (643/946) in the supplemented group showed positive anthropometric outcomes. The distribution of negative and non-positive (supplemented group) outcomes is presented in S3 Table.

In the unadjusted analysis, five exposures were associated with a higher risk of negative anthropometric outcomes among the unsupplemented group: lower age (months), age < 2 years, lower body weight (kg), lower height (cm), and being breastfed (Table 2). Each additional yearly increase of age was associated with approximately a 16% lower risk of a negative anthropometric outcome (RR: 0.84; 95% CI: 0.71, 0.99; p = 0.043). Children under 2 years of age had a 59% higher risk than those aged 2 years or older (RR 1.59; 95% CI: 1.10, 2.26; p = 0.011). Each additional kilogram of body weight was associated with a 13% lower risk (RR 0.87; 95% CI 0.77, 0.99; p = 0.034), and each additional centimetre of height with a 3% lower risk (RR 0.97; 95% CI: 0.951, 0.997; p = 0.031). Children who were currently breastfeeding had a 49% higher risk compared with non-breastfed children (RR 1.49; 95% CI: 1.04, 2.11; p = 0.026). None of the associations remained significant in the adjusted analysis (Fig 2). The full list of baseline exposures assessed is presented in S4 Table.

**Table 2:** Exposures associated with negative anthropometric outcomes among severely underweight children not receiving nutritional supplementation (Unadjusted analysis).

Exposure	Number at risk	Events	Crude RR (95% CI)	P-value
**Demographic**				
Age (years)	432	151	0.84 (0.71, 0.99)	0.043
Age Group				
Over 2 Years	350	110	1.00 (Reference)	
Under 2 Years	82	41	1.59 (1.1, 2.26)	0.011
**Anthropometry and Body Composition**				
Weight (kg)	432	151	0.87 (0.77, 0.99)	0.034
Height (cm)	432	151	0.97 (0.951, 0.997)	0.031
**Infant and Young Child Feeding**				
Currently Breastfeeding				
No	341	108	1.00 (Reference)	
Yes	91	43	1.49 (1.04, 2.11)	0.026

CI = confidence interval; RR = relative risk.

**Fig 2:**
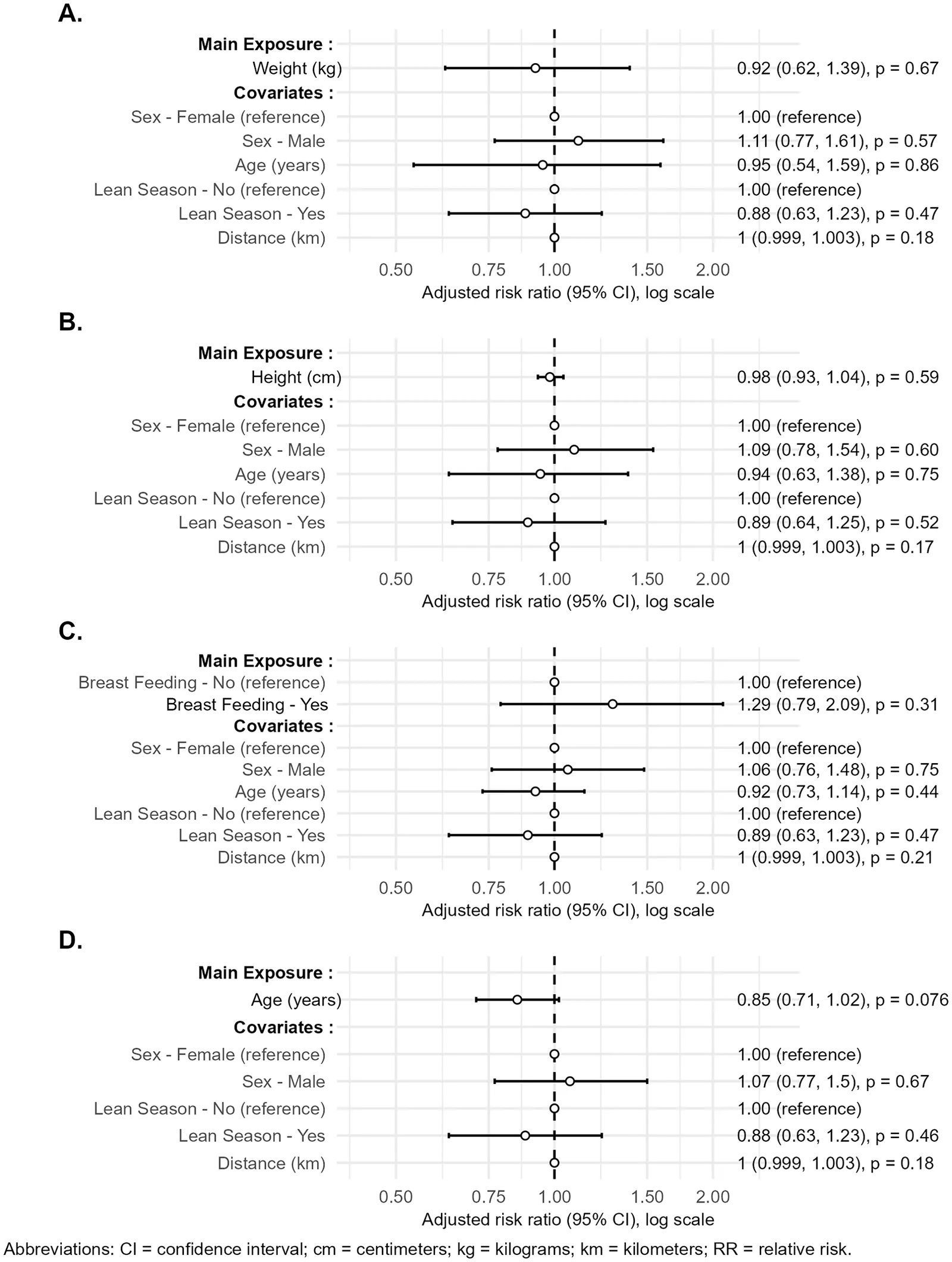
Adjusted associations with negative anthropometric outcomes among unsupplemented children. Each panel presents a separate adjusted model in which the baseline characteristic named is the main exposure: (A) weight (kg), (B) height (cm), (C) currently breastfeeding, and (D) age (years). CI, confidence interval; RR, risk ratio; RUTF, ready-to-use therapeutic food.

Among those receiving nutritional supplementation, the unadjusted analyses identified one statistically significant association with positive anthropometric outcomes: illness (Table 3). Children with a recent illness had a 23% higher likelihood of a positive anthropometric outcome during nutrition treatment than those without a recent illness (RR 1.23; 95% CI 1.05, 1.43; p = 0.01). The full list of baseline exposures (unadjusted analyses) is presented in S5 Table.

**Table 3:** Exposures associated with positive anthropometric outcomes among severely underweight children receiving nutritional support over a 12-week follow-up.

Exposure	Number at risk	Events	Crude RR (95% CI)	P-value
**Clinical**				
Recent Illness				
No	539	334	1.00 (Reference)	
Yes	407	309	1.23 (1.05, 1.43)	0.010
**Infant and Young Child Feeding**				
Currently Breastfeeding				
No	710	504	1.00 (Reference)	
Yes	236	139	0.83 (0.685, 0.998)	0.051

CI = confidence interval; RR = relative risk.

The association remained significant in the adjusted analyses, with recent illness being the only statistically significant predictor. Children with a recent illness had a 22% higher probability of positive anthropometric outcomes during nutritional supplementation than those without illness (RR = 1.22; 95% CI = 1.03, 1.43; p = 0.019) (Fig 3).

**Fig 3:**
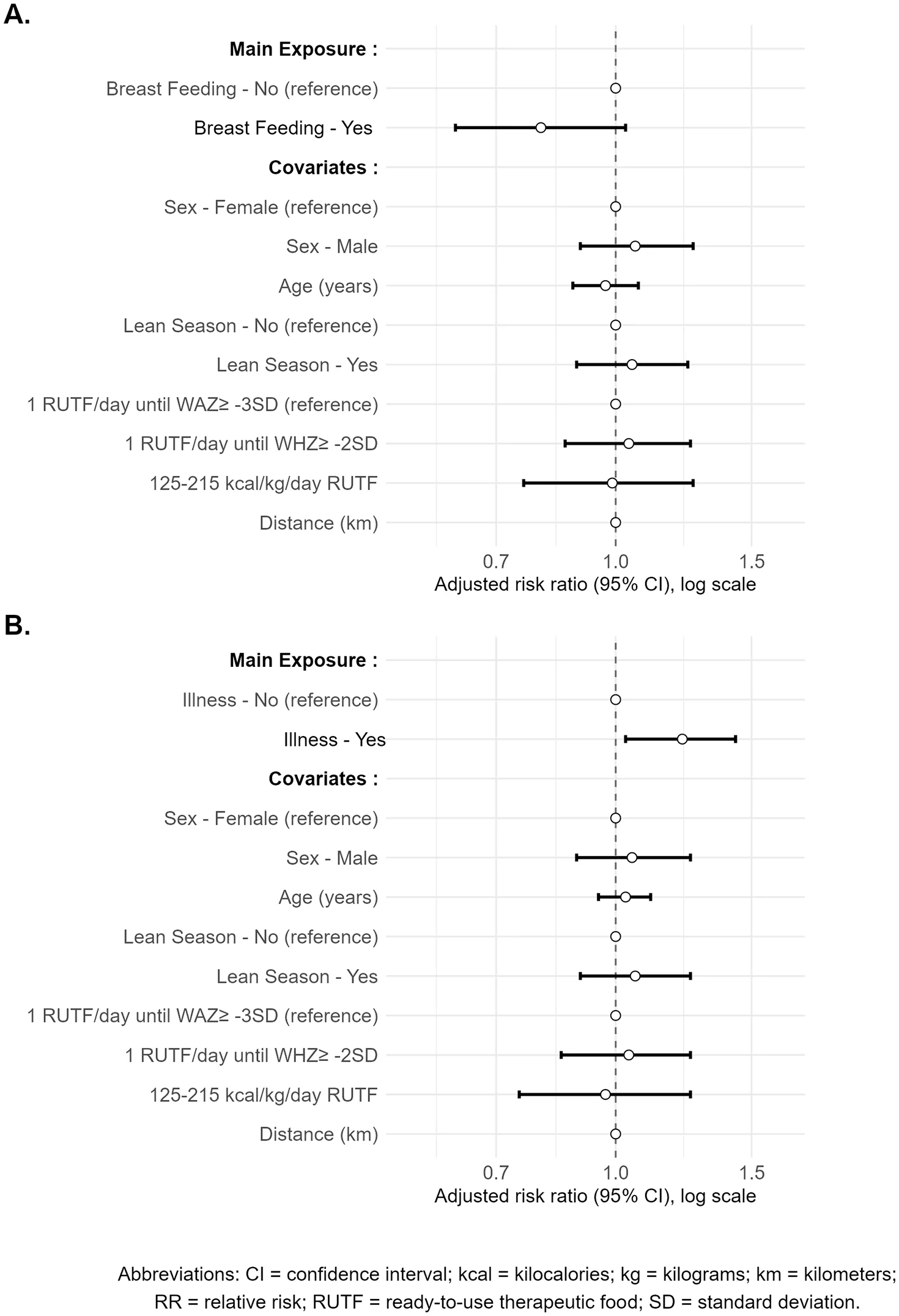
Adjusted associations with positive anthropometric outcomes among supplemented children.outcomes among supplemented children. Each panel presents a separate adjusted model in which the baseline characteristic named is the main exposure: (A) currently breastfeeding and (B) recent illness. CI, confidence interval; RR, risk ratio; RUTF, ready-to-use therapeutic food.

We did not identify any baseline characteristic that was associated with both a negative anthropometric outcome (in the absence of supplementation) and a positive anthropometric outcome (with supplementation).

### Sensitivity analyses

Sensitivity analyses showed that the association estimates were generally consistent with the primary analysis (S6 and S7 Tables). Among unsupplemented children, age was not statistically significant under the primary outcome definition (adjusted RR 0.85; 95% CI: 0.71,1.02), but became significant when more stringent thresholds for a negative outcome were applied (RR 0.78; 95% CI: 0.64, 0.94 for ΔWAZ < −0.05; RR: 0.71; 95% CI: 0.58, 0.88 for ΔWAZ <−0.10). Estimates for baseline weight, height, and breastfeeding remained non-significant across the sensitivity analyses. Among supplemented children, recent illness remained significantly associated with a positive anthropometric outcome across all sensitivity analyses, with adjusted RRs ranging from 1.18 to 1.43.

#### Additional diagnostic analyses.

Missingness for each candidate exposure, by group and by outcome status, is presented in S8 Table and S9 Table. The follow-up cascade for each group is shown in S10 Table. In a post-hoc analysis restricted to currently breastfed children in the unsupplemented group, most of those with a negative anthropometric outcome were aged 12–23 months (S11 Table).

## Discussion

To our knowledge, this is the first study to examine factors associated with negative anthropometric outcomes among severely underweight children not receiving nutritional supplementation, as well as factors associated with positive anthropometric outcomes among those receiving supplementation. While several baseline characteristics were associated with negative anthropometric outcomes, only one (recent illness) was associated with positive anthropometric outcomes, and none were associated with both. Although the adverse outcomes of interest included severe illness and death, fewer than 1% of children experienced death, and fewer than 2% experienced severe illness. Given the low incidence of mortality and severe morbidity in our sample, these negative outcomes are largely anthropometric outcomes.

The lack of factors simultaneously associated with both negative and positive anthropometric outcomes in this study highlights the paucity of evidence on effective strategies for identifying which subgroup of severely undernourished children is at risk of negative outcomes in the absence of nutritional supplementation and is likely to benefit from nutritional supplementation. For example, the 2023 WHO recommendation on moderate wasting-targeting strategies was based on factors associated with negative health outcomes [3], but it is unclear whether nutritional supplementation improves outcomes [23,24]. In the present study, the absence of a shared prognostic marker does not imply that supplementation confers no benefit. It may be that children most likely to experience slowed growth (negative anthropometric outcomes) without supplementation would at least maintain normal growth with supplementation, even if they did not catch up in growth (attaining positive anthropometric outcomes). This would need to be evaluated through an analysis comparing similar outcomes among comparable groups, which was not the approach in this study. The present within-group design cannot estimate individual benefit from supplementation, because the same baseline characteristic was not compared across supplemented and unsupplemented children under a common outcome definition.

Among the unsupplemented group, children with lower body weight and height, young age, and specifically children under two years, and children who were currently breastfeeding, had a higher risk of negative anthropometric outcomes. However, after adjustment for age and other covariates, these associations were no longer significant, suggesting that these factors primarily reflect correlations with young age. This finding is similar to other studies on undernourished children that reported that, compared to children aged two years and above, children under the age of two face a higher risk of poor health outcomes, including recurrent/persistent malnutrition and mortality [9]. However, other studies have also reported comparable mortality risk across age groups [25]. Further studies are therefore needed to identify the contexts in which younger children are at greater risk of adverse outcomes and to determine effective interventions that can reduce the age-associated risk. In particular, the observed association between breastfeeding and negative anthropometric outcomes is therefore unlikely to be causal and may instead reflect reverse causality, whereby mothers prolong breastfeeding in response to poor child growth rather than breastfeeding causing growth faltering [26]. Prior studies show breastfeeding improves survival among children with low WAZ, and children nearing cessation have higher WAZ than those who continue [26]. Among currently breastfed children in the unsupplemented group with a negative anthropometric outcome, most were aged 12–23 months. However, exclusive breastfeeding duration and age at introduction of complementary foods were not recorded, so we could not formally test whether these outcomes cluster around the weaning transition.

From a programmatic perspective, identifying children at greatest vulnerability does not necessarily require establishing causality. This study, therefore, did not seek to determine underlying causal mechanisms but rather identified prognostic factors of health outcomes among underweight children. If young age consistently identifies children at higher risk of poor outcomes, it may serve as a practical proxy for targeting additional support among children who do not meet current treatment criteria but remain biologically vulnerable. In this instance, however, additional nutritional support was not associated with a higher likelihood of positive anthropometric outcomes among children under the age of two.

Interestingly, ill children were more likely to have a positive anthropometric outcome from supplementation in both adjusted and unadjusted analyses. This finding is biologically plausible and may reflect greater potential for catch-up in weight among children recovering from acute illness [27]. Episodes of illness are commonly associated with transient catabolic states due to reduced intake and increased metabolic demands [28]. Nutritional supplementation during recovery may therefore result in greater catch-up ponderal growth than in children who are relatively metabolically stable [29,30]. In addition, referral for and receipt of medical treatment may have facilitated recovery from illness, thereby improving appetite, nutrient utilisation, and the effectiveness of supplementation. This association suggests that children transitioning from acute illness had a higher likelihood of a positive (largely anthropometric) prognostic outcome, consistent with post-illness rebound rather than enhanced responsiveness to supplementation [31]; however, it is highly speculative and warrants further study. Accordingly, recent illness is better regarded as a possible marker of post-illness anthropometric rebound, in which transiently reduced baseline weight allows greater scope for weight gain during recovery, than as evidence of enhanced responsiveness to RUTF.

Although the RUTF dose was included as a confounder rather than an exposure in the models restricted to children receiving nutritional supplementation, there was no association between RUTF dose and health outcomes, suggesting that the potential positive effects of supplementation were not dependent on RUTF dose. Similarly, findings from the primary RCT indicated that WAZ changes were similar between children in the simplified and standard-of-care arms (Kangas et al. in press). This aligns with the evolution of WHO guidance, which in 2023 reduced RUTF doses for treatment of severe wasting from 150-220 kcal/kg/day to 150–180 kcal/kg/day in the initial severe phase and to 100–130 kcal/kg/d in the moderate phase [3,32,33].

The cohort comprised 57% boys. Although not statistically significant, this is consistent with the higher burden of underweight among boys documented globally [34] and in Mali's national SMART surveys, where underweight prevalence is consistently higher in boys than in girls: 23% vs 19% in 2024; 20% vs 16% in 2019 [35,36]. Enrolment was based on anthropometric eligibility, with no selection by age or sex, so the male predominance reflects the underlying epidemiology rather than the sampling design.

Several aspects warrant consideration when interpreting this study. First, by design, children with MUAC <125 mm were excluded to focus on those missed by current MUAC-based treatment criteria; this was a deliberate programmatic choice rather than a limitation, as the parent study aimed to test the addition of WAZ < -3 to MUAC-based treatment criteria. Similarly, this secondary analysis aimed to identify risk factors in this subpopulation of severely underweight children excluded from MUAC-based programmes. Additionally, the cut-offs for WAZ changes included in the composite measure to detect positive anthropometric outcomes (≥ 0.15) or adversity (< 0) were arbitrarily chosen and may not reflect true positive or negative anthropometric outcomes. These thresholds should not be interpreted as clinically validated markers of benefit or harm, and small WAZ changes over 12 weeks may partly reflect measurement error, hydration, illness recovery, or regression to the mean. The cut-offs were based on the common assumption that a decline in the severity of undernutrition, as measured by WAZ or MUAC, among children under 5 years is usually associated with reductions in mortality and adverse outcomes. However, the association between anthropmetry and mortality is only suggestive, and improvements in negative ponderal anthropometric outcomes may also be driven by micronutrient status and immunity rather than by macronutrient status alone. Additionally, because this was a secondary analysis, it may have lacked sufficient statistical power due to an inadequate sample size for some exposures; for example, bioelectrical impedance analysis measures used to estimate FFM and FM were collected from only a subset of children in the parent study. Because body-composition measures were available for only about one-third of children, we restricted models including these variables in sensitivity analyses; such missingness may relate to site, operational capacity, illness status, or child cooperation. We examined missingness for each candidate exposure by group and cross-tabulated it by outcome status within each group to assess whether missingness was related to outcome. Body-composition measures (only when BIA machines were available) and malaria rapid diagnostic testing (only among children with fever) were the only variables with substantial missingness (each performed in a subset), whereas haemoglobin and anaemia status were missing for approximately 1% of children and all remaining candidate exposures were complete in both groups. Another limitation is that severe illness events and deaths were uncommon in this cohort (with three deaths overall). As a result, both the positive and negative composite outcomes were largely driven by anthropometric changes rather than clinical events. This means that the identified factors should primarily be interpreted as associated with ponderal anthropometric deterioration or improvement. More broadly, because many candidate exposures were examined and were screened by univariable significance before adjustment, the analysis is exploratory and the reported associations should be regarded as hypothesis-generating rather than confirmatory. Isolated nominal significance may reflect false-positive discovery, while loss of significance after adjustment may reflect collinearity, over-adjustment, or limited power rather than a true absence of prognostic signal. Finally, the parent trial recorded only current breastfeeding status at enrolment, and neither exclusive breastfeeding duration nor age at introduction of complementary foods; we could therefore not test whether negative anthropometric outcomes among breastfed children cluster around the transition to complementary feeding.

The key strength of this study is that it used data from an RCT, which provided a comprehensive dataset encompassing a wide range of baseline variables, from individual-level to household-level characteristics. Another strength is the robustness of the analyses, with results largely consistent with the sensitivity analyses. These findings should be replicated in larger cohorts, ideally from higher-mortality settings, where death and severe morbidity occur more frequently, and the composite outcomes would be less dominated by short-term anthropometric change.

## Conclusion

Among severely underweight children who do not meet the MUAC threshold for wasting treatment using the simplified protocol, we found no single characteristic that predicted both a high risk of negative anthropometric outcomes in the absence of nutritional treatment and a high likelihood of positive anthropometric outcomes in the presence of nutritional treatment. Age below two years was associated with a higher likelihood of adverse anthropometric outcomes among untreated children, and recent illness was associated with improved anthropometric outcomes among supplemented children. These findings highlight the difficulty of prioritising treatment among severely underweight children.

## Supporting information

S1 CONSORT Checklist. (DOCX)

S1 Table Description of variables of interest. (DOCX)

S2 Table Additional baseline characteristics among severely underweight children. (DOCX)

S3 Table Outcome composition and reasons for referral or inpatient care among severely underweight children, by group. (DOCX)

S4 Table Full list of exposures associated with negative outcomes among severely underweight children not receiving nutritional support. (DOCX)

S5 Table Full list of exposures associated with positive anthropometric outcomes among children receiving nutritional support (unadjusted analysis). (DOCX)

S6 Table Sensitivity analyses: adjusted risk ratios (95% CI) for the principal baseline predictors under alternative outcome definitions and analytic approaches among unsupplemented children (negative outcomes). (DOCX)

S7 Table Sensitivity analyses: adjusted risk ratios (95% CI) for the principal baseline predictors under alternative outcome definitions and analytic approaches among supplemented children (positive outcomes). (DOCX)

S8 Table Missingness of candidate exposures by group. (DOCX)

S9 Table Missingness of candidate exposures by outcome status, within group. (DOCX)

S10 Table Follow-up cascade and outcome assignment, by exposure group. (DOCX)

S11 Table Age distribution of currently breastfed children with a negative anthropometric outcome (unsupplemented group). (DOCX)

## References

[1] Black RE, Victora CG, Walker SP, Bhutta ZA, Christian P, de Onis M, et al. Maternal and child undernutrition and overweight in low-income and middle-income countries. Lancet. 2013;382(9890):427–51. doi: 10.1016/S0140-6736(13)60937-X 23746772

[2] Olofin I, McDonald CM, Ezzati M, Flaxman S, Black RE, Fawzi WW, et al. Associations of suboptimal growth with all-cause and cause-specific mortality in children under five years: a pooled analysis of ten prospective studies. PLoS One. 2013;8(5):e64636. doi: 10.1371/journal.pone.0064636 23734210 PMC3667136

[3] World Health Organisation. WHO guideline on the prevention and management of wasting and nutritional oedema (acute malnutrition) in infants and children under 5 years. 2023. https://files.magicapp.org/guideline/a3fe934f-6516-460d-902f-e1c7bbcec034/published_guideline_7330-1_1.pdf 38498638

[4] Global Health Observatory. Underweight prevalence among children under 5 years of age (%) (model-based estimates). 2022. [cited in 2026 Aug 21]. https://www.who.int/data/gho/data/indicators/indicator-details/GHO/gho-jme-underweight-prevalence

[5] World Health Organisation. Fact sheets - Malnutrition. 2024. [cited in 2026 July 1]. https://www.who.int/news-room/fact-sheets/detail/malnutrition

[6] World Health Organisation. Underweight prevalence among children under 5 years of age (%) (model-based estimates). 2025. [cited in 2026 July 1]. https://www.who.int/data/gho/data/indicators/indicator-details/GHO/gho-jme-underweight-prevalence

[7] Olofin I, McDonald C hristine M, Majid E, Flaxman S, Black RE, Fawzi WW, et al. Associations of suboptimal growth with all-cause and cause-specific mortality in children under five years: A pooled analysis of ten prospective studies. PLoS ONE. 2013. doi: 10.1371/journal.pone.0064636 PMC3667136 23734210

[8] Khara T, Myatt M, Sadler K, Bahwere P, Berkley JA, Black RE, et al. Anthropometric criteria for best-identifying children at high risk of mortality: a pooled analysis of twelve cohorts. Public Health Nutr. 2023;26(4):803–19. doi: 10.1017/S136898002300023X 36734049 PMC10131149

[9] Thurstans S, Wrottesley SV, Fenn B, Khara T, Bahwere P, Berkley JA, et al. Anthropometric deficits and the associated risk of death by age and sex in children aged 6-59 months: A meta-analysis. Matern Child Nutr. 2023;19(1):e13431. doi: 10.1111/mcn.13431 36164997 PMC9749608

[10] Kangas ST, Ouédraogo CT, Tounkara M, Ouoluoguem B, Coulibaly IN, Haidara A, et al. Nutritional treatment of children 6–59 months with severely low weight-for-age z-score: a study protocol for a 3-arm randomized controlled trial. Trials. 2024;25(1):30. doi: 10.1186/s13063-023-07890-0 PMC10773065 38191436

[11] Kangas ST, Marron B, Tausanovitch Z, Radin E, Andrianarisoa J, Dembele S, et al. Effectiveness of acute malnutrition treatment at health center and community levels with a simplified, combined protocol in Mali: an observational cohort study. Nutrients. 2022;14(22):4923. doi: 10.3390/nu14224923 36432609 PMC9699530

[12] Satav AR, Dani VS, Pendharkar JS, Satav KA, Raje D, Jain D, et al. Locally prepared therapeutic food for treatment of severely underweight children in rural India: an interventional prospective controlled community-based study with long follow-up:—‘SAMMAN’ Trial. Nutrients. 2024;16(17):2872. doi: 10.3390/nu16172872 PMC11397721 39275188

[13] WHO. WHO guideline on the prevention and management of wasting and nutritional oedema (acute malnutrition) in infants and children under 5 years. 2023. https://files.magicapp.org/guideline/3a21f239-4772-4c05-bf23-ca6d1d08f086/published_guideline_6412-1_0.pdf 38498638

[14] FEW.NET. Mali Food Security Outlook. Washington, DC, USA: FEW.NET; 2021. https://fews.net/west-africa/mali/food-security-outlook/october-2021/print

[15] FEWS.NET. Mali Food Security Outlook. Washington, DC, USA: FEWS.NET; 2018. https://fews.net/west-africa/mali/food-security-outlook/december-2018/print

[16] Institut National de la Statistique du Mali INSTAT. Enquête nationale nutritionnelle anthropométrique et de mortalité rétrospective Mali (SMART). 2023. [cited in 2024 Oct 14]. https://www.instat-mali.org/fr/publications/enquete-nationale-nutritionnelle-anthropometrique-et-de-mortalite-retrospective-mali-smart

[17] Owusu DN, Duah HO, Dwomoh D, Alhassan Y. Prevalence and determinants of diarrhoea and acute respiratory infections among children aged under five years in West Africa: evidence from demographic and health surveys. Int Health. 2024;16(1):97–106. doi: 10.1093/inthealth/ihad046 37387288 PMC10759282

[18] Malaria in Mali: Statistics & Facts. Severe Malaria Observatory. 2023. [cited 2026 Aug 4]. https://www.severemalaria.org/countries/mali

[19] DHS. Demographic Health Survey Mali 2021. DHS; 2021. https://dhsprogram.com/methodology/survey/survey-display-613.cfm

[20] Instat I de la S, Cellule de Planification et de Statistique du Secteur Santé DS et P de la FCDP, ICF. Enquête démographique et de santé au Mali 2023–2024. 2025. https://dhsprogram.com/publications/publication-FR394-DHS-Final-Reports.cfm

[21] R Core Team. R: A Language and Environment for Statistical Computing. R Foundation for Statistical Computing. 2024 [cited in 2026 October 30]. https://www.R-project.org/

[22] Food and Nutrition Technical Assistance III Project (FANTA). Household Hunger Scale (HHS): Indicator Definition and Measurement Guide. 2018. [cited in 2025 July 6]. https://www.fantaproject.org/monitoring-and-evaluation/household-hunger-scale-hhs

[23] Trehan I, Bandsma RHJ, Borg B, Castro MC, Golden K, Thompson DS, et al. Infants and children 6-59 months of age with moderate wasting: evidence gaps identified during WHO guideline development. BMJ Glob Health. 2025;10(Suppl 5):e017228. doi: 10.1136/bmjgh-2024-017228 40803704 PMC12352201

[24] Potani I, Tausanovitch Z, Heymsfield G, Ritz C, Cazes C, James PT, et al. Applying WHO prioritization criteria for moderate wasting: programmatic implications. Matern Child Nutr. 2026;22(1):e70152. doi: 10.1111/mcn.70152 41353631

[25] Schwinger C, Group WHORSWG. Infant- and child-level predictors of mortality in low-resource settings: the WHO child mortality risk stratification multi-country pooled cohort. medRxiv. 2024;2024.07.06.24309988. doi: 10.1101/2024.07.06.24309988 40288395

[26] Briend A, Bari A. Breastfeeding improves survival, but not nutritional status, of 12-35 months old children in rural Bangladesh. Eur J Clin Nutr. 1989;43(9):603–8. 2606091

[27] Bourdon C, Diallo AH, Mohammad Sayeem Bin Shahid AS, Khan MA, Saleem AF, Singa BO, et al. Childhood growth during recovery from acute illness in Africa and South Asia: a secondary analysis of the childhood acute illness and nutrition (CHAIN) prospective cohort. EClinicalMedicine. 2024;70:102530. doi: 10.1016/j.eclinm.2024.102530 38510373 PMC10950691

[28] Liu H, Wang S, Wang J, Guo X, Song Y, Fu K, et al. Energy metabolism in health and diseases. Signal Transduct Target Ther. 2025;10(1):69. doi: 10.1038/s41392-025-02141-x 39966374 PMC11836267

[29] van der Kam S, Swarthout T, Niragira O, Froud A, Sompwe EM, Mills C, et al. Ready-to-use therapeutic food for catch-up growth in children after an episode of Plasmodium falciparum malaria: an open randomised controlled trial. PLoS One. 2012;7(4):e35006. doi: 10.1371/journal.pone.0035006 22558108 PMC3338820

[30] Hoare S, Poppitt SD, Prentice AM, Weaver LT. Dietary supplementation and rapid catch-up growth after acute diarrhoea in childhood. Br J Nutr. 1996;76(4):479–90. doi: 10.1079/bjn19960057 8942357

[31] NUTRIMAM Study Team. A multi-country, randomized trial of three nutritional supplements on short-term and sustained anthropometric recovery in children 6-24 months of age with moderate wasting and acute illnesses: the NUTRIMAM study protocol. Trials. 2024;25(1):738. doi: 10.1186/s13063-024-08390-5 39501315 PMC11536563

[32] World Health Organisation. WHO guideline: updates on the management of severe acute malnutrition in infants and children. World Health Organization; 2013. https://www.who.int/publications/i/item/9789241506328 24649519

[33] Likoswe BH, Chimera-Khombe B, Patson N, Selemani A, Potani I, Phuka J, et al. A systematic review on the optimal dose and duration of Ready-to-Use Therapeutic Food (RUTF) for 6-59-Month-Old Children with Severe Wasting or Oedema. Nutrients. 2023;15(7):1750. doi: 10.3390/nu15071750 37049590 PMC10096907

[34] Thurstans S, Opondo C, Seal A, Wells J, Khara T, Dolan C, et al. Boys are more likely to be undernourished than girls: a systematic review and meta-analysis of sex differences in undernutrition. BMJ Glob Health. 2020;5(12):e004030. doi: 10.1136/bmjgh-2020-004030 33328202 PMC7745319

[35] Institut National de la Statistique du Mali INSTAT. Enquête nationale nutritionnelle anthropométrique et de mortalité rétrospective Mali (SMART). 2019. https://www.instat-mali.org/fr/publications/enquete-nationale-nutritionnelle-anthropometrique-et-de-mortalite-retrospective-mali-smart

[36] SMART Surveys. Enquête nutritionnelle anthropométrique et de mortalité retrospective au Mali - 14e edition, août - septembre 2024 - Mali. 2024. [cited in 2026 Aug 12]. https://reliefweb.int/report/mali/enquete-nutritionnelle-anthropometrique-et-de-mortalite-retrospective-au-mali-14e-edition-aout-septembre-2024

