# Supplementary material for: Identifying risk and response to nutritional supplementation in severely underweight children missed by standard treatment criteria in Mali: A secondary prognostic analysis of data from a randomised controlled trial
Source: PLOS Glob Public Health. 2026 Nov 12;6(11):e0007525. doi: 10.1371/journal.pgph.0007525 (PMC13759120; doi:10.1371/journal.pgph.0007525)
Supplement: Supplementary file 2 [file pgph.0007525.s002.docx]

**S1 Table: Description of variables of interest**

| **Domain** | **Variable** | **Description** | **Type/structure (Units)** | **Categories / Levels** |
| --- | --- | --- | --- | --- |
| Demographic | Age at enrolment | Age of participant at enrolment | Continuous (months) | - |
|  | Age group | Age category | Binary | < 24 months; ≥ 24 months |
|  | Sex | Sex of participant | Binary | Female; Male |
| Anthropometry and body composition | Weight | Body weight | Continuous (kg) | - |
|  | Height | Height | Continuous (cm) |  |
|  | WHZ at enrolment | Weight-for-height z-score | Continuous (z-score) |  |
|  | Wasting | Presence of wasting | Binary | Wasting (WHZ< −2 SD); No wasting (WHZ≥ −2 SD) |
|  | WAZ at enrolment | Weight-for-age z-score | Continuous (z-score) |  |
|  | HAZ at enrolment | Height-for-age z-score | Continuous (z-score) |  |
|  | Stunting | Presence of stunting | Binary | Stunting (HAZ< −2 SD); No stunting (HAZ≥ −2 SD) |
|  | MUAC | Mid-upper arm circumference | Continuous (mm) |  |
|  | Fat-free mass (FFM) |  | Continuous (kg) |  |
|  | Fat mass (FM) | Body weight of fat mass | Continuous (kg) |  |
|  | Fat-free mass index (FFMI) | FFM / height² | Continuous (kg/m²) |  |
|  | Fat mass index (FMI) | FM / height² | Continuous (kg/m²) |  |
|  | Subscapular skinfold z-score | Z-score for subscapular skinfold thickness | Continuous (z-score) |  |
|  | Triceps skinfold Z-score | Z-score for triceps skinfold thickness | Continuous (z-score) |  |
| Clinical | Hemoglobin (Hb) | Hemoglobin concentration | Continuous (g/dL) |  |
|  | Anemia | Anemia status based on haemoglobin | Binary | Anemic (Hb< 11 g/dL); Not anemic (Hb≥ 11 g/dL) |
|  | Recent illness | Illness in past 2 weeks (caregiver report, clinical exam, or malaria RDT) | Binary | Yes, no |
|  | Malaria test result | Malaria Rapid Diagnostic Test result | Binary | Positive; Negative |
|  | Vaccines up to date | Completeness of vaccinations | Binary | Complete; Not complete |
| Infant and young child feeding | Currently breastfeeding | Breastfeeding at time of assessment | Binary | Yes, no |
|  | Breastfeeding frequency | Number of breastfeeding episodes in 24h | Continuous (count) |  |
|  | Previous day breastfeeding history | Number of breastfeeding episodes on previous day | Continuous (count) |  |
|  | Dietary diversity score | Number of food groups consumed in previous 24h | Continuous (count) |  |
|  | Minimum dietary diversity | Meets ≥ 5 food groups | Binary | Yes, no |
|  | Child went hungry previous day | Whether child went hungry yesterday | Binary | Yes, no |
|  | Frequency child went hungry | Number of times hungry yesterday | Continuous (count) |  |
|  | Self-feeding | Child self-feeds | Binary | Yes, no |
|  | Went to bed hungry | Whether child went to bed hungry | Binary | Yes, no |
|  | Dosage of therapeutic food | Intervention type | Categorical | 1. No intervention;  2. 1 sachet (500 kcal) RUTF/day until WAZ ≥ −3;  3. 1 sachet (500 kcal) RUTF/day until WHZ ≥ −2;  4. 125-215 kcal/kg/day RUTF until WHZ ≥ −2; |
| Caregiver and household characteristics  Caregiver and household characteristics | Caregiver age | Age of primary caregiver | Continuous (years) |  |
|  | Caregiver education | Highest education level of caregiver | Categorical | No education; Primary or lower; Secondary or higher |
|  | Household head occupation | Occupation of household head | Binary | Formal; Informal |
|  | Household head employment | Employment status of household head | Binary | Employed; Unemployed |
|  | Household size | Number of household members | Continuous (count) |  |
|  | Household size category | Size category based on quantiles | Binary | Large (≥ 4 children); Small–medium (1–3 children) |
|  | Household hunger score | Household hunger severity score | Continuous (score) |  |
|  | Food shortage frequency | Number of food shortage episodes | Continuous (count) |  |
|  | Number of children in household | Count of children in household | Continuous (count) |  |
|  | Floor material | Type of household floor | Binary | Cement; basic (mud/dung) |
|  | Socioeconomic status score | SES score | Continuous (score) |  |
|  | Socioeconomic status | SES category | Categorical | Low; Medium; High |
|  | Water source | Type of water source | Binary | Improved (tap/borehole); Unimproved (open water source e.g. river) |
|  | Treatment of drinking water | Water treated before drinking | Binary | Yes (antimicrobial processing), no |
|  | Electricity access | Access to electricity | Binary | Yes, no |
|  | Latrine use | Use of latrine | Binary | Yes, no |
|  | Season at enrolment | Lean season at enrolment | Binary | Yes (February-October enrolment, no (November-January enrolment) |
|  | Distance to health facility | Distance from village to nearest health facility | Continuous (km) |  |
|  | Received food aid | Receipt of external food aid | Binary | Yes, no |
| Outcome variables | Positive outcome | Composite positive outcome among supplemented children | Binary | WAZ change ≥ 0.15 SD AND MUAC ≥ 125 mm AND not hospitalized AND alive: |
|  | Negative outcome | Composite negative outcome among unsupplemented children | Binary | WAZ change < 0.0 SD, OR MUAC < 125 mm, OR hospitalized |
| *Abbreviations: WHZ, weight-for-height z-score; WAZ, weight-for-age z-score; HAZ, height-for-age z-score; MUAC, mid-upper arm circumference; FFM, fat-free mass; FM, fat mass; FFMI, fat-free mass index; FMI, fat mass index; Hb, haemoglobin; SD, standard deviation; RDT, rapid diagnostic test; RUTF, ready-to-use therapeutic food; kcal, kilocalories; SES, socioeconomic status. Continuous variables are presented with units as specified; categorical and binary variables are defined by listed categories* | | | | |
