# Supplementary material for: Identifying risk and response to nutritional supplementation in severely underweight children missed by standard treatment criteria in Mali: A secondary prognostic analysis of data from a randomised controlled trial
Source: PLOS Glob Public Health. 2026 Nov 12;6(11):e0007525. doi: 10.1371/journal.pgph.0007525 (PMC13759120; doi:10.1371/journal.pgph.0007525)
Supplement: Supplementary file 3 [file pgph.0007525.s003.docx]

**S2 Table Additional baseline characteristics in the analysis of positive and negative anthropometric outcomes**

|  | **Unsupplemented group** | **Supplemented group** | **All children** | |
| --- | --- | --- | --- | --- |
| **Characteristic at Enrolment** | **Median[IQR] / n(%)** | **Median[IQR] / n(%)** | **Median[IQR] / n(%)** | **N(%)** |
| **Total (n) %** | **432 (100%)** | **946 (100%)** | **1378 (100%)** |  |
| **Anthropometry and Body Composition** |  | | | |
| FFMI (kg/m2)† | (155) 10.7 [9.9,11.8] | (294) 10.6 [9.7,11.5] | 10.6 [9.8,11.6] | 449 (32.6%) |
| FMI (kg/m2)† | (149) 3.4 [2.3,4.0] | (288) 3.0 [2.3,3.7] | 3.1 [2.3,3.9] | 437 (31.7%) |
| Triceps skinfold thickness (mm) | 6.8 [6.3,7.7] | 6.7 [5.9,7.5] | 6.7 [6.1,7.5] | 1378 (100.0%) |
| Subscapular skinfold thickness (mm) | 5.3 [4.7,5.9] | 5.1 [4.5,5.7] | 5.1 [4.5,5.7] | 1378 (100.0%) |
| **Clinical** |  | | | |
| Positive Malaria RDT on enrolment† | 121/160 (76%) | 251/334 (75%) | 372 (75%) | 494 (35.8%) |
| Vaccines Up To Date | 92 (21%) | 220 (23%) | 312 (23%) | 1378 (100.0%) |
| **Infant and Young Child Feeding** |  | | | |
| **Breastfeeding Frequency** |  | | | |
| 1-2 | 2 (0%) | 4 (0%) | 6 (0%) | 327 (23.7%) |
| 3-4 | 23 (5%) | 48 (5%) | 71 (5%) | 327 (23.7%) |
| 5+ | 66 (15%) | 184 (19%) | 250 (18%) | 327 (23.7%) |
| **Going To Bed Hungry Frequency (Past Month)** |  | | | |
| Never | 401 (93%) | 881 (93%) | 1282 (93%) | 1378 (100.0%) |
| Rarely (1-2 times) | 20 (5%) | 39 (4%) | 59 (4%) | 1378 (100.0%) |
| Sometimes (3-10 times) | 11 (3%) | 24 (3%) | 35 (3%) | 1378 (100.0%) |
| Often (More than 10 times) | -- | 2 (0%) | 2 (0%) | 1378 (100.0%) |
| **Caregiver and Household Characteristics** |  | | | |
| Care giver Age (years)† | (16) 29.5 [22.8,35.2] | (42) 31.5 [26,38.2] | 30.5 [25.2,36] | 58 (4.2%) |
| Caregiver education - Primary or Lower | 86 (20%) | 207 (22%) | 293 (21%) | 1378 (100.0%) |
| **Food Shortages Frequency (Past Month)** |  | | | |
| Never | 394 (91%) | 854 (90%) | 1248 (91%) | 1378 (100.0%) |
| Rarely (1-2 times) | 27 (6%) | 54 (6%) | 81 (6%) | 1378 (100.0%) |
| Sometimes (3-10 times) | 9 (2%) | 34 (4%) | 43 (3%) | 1378 (100.0%) |
| Often (More than 10 times) | 2 (0%) | 4 (0%) | 6 (0%) | 1378 (100.0%) |
| Floor material- Cement | 33 (8%) | 76 (8%) | 109 (8%) | 1378 (100.0%) |
| Improved Water Source | 265 (61%) | 629 (66%) | 894 (65%) | 1378 (100.0%) |
| Treated water access | 106 (25%) | 226 (24%) | 332 (24%) | 1378 (100.0%) |
| Electricity Access | 108 (25%) | 280 (30%) | 388 (28%) | 1378 (100.0%) |
| Latrine use | 199 (46%) | 528 (56%) | 727 (53%) | 1378 (100.0%) |
| Lean season | 208 (48%) | 494 (52%) | 702 (51%) | 1378 (100.0%) |
| **Socioeconomic Status (SES) Tertiles** |  | | | |
| Low SES | 154 (36%) | 306 (32%) | 460 (33%) | 1378 (100.0%) |
| Mid SES | 135 (31%) | 324 (34%) | 459 (33%) | 1378 (100.0%) |
| High SES | 143 (33%) | 316 (33%) | 459 (33%) | 1378 (100.0%) |
| Abbreviations: FFM, fat-free mass; FFMI, fat-free mass index; FM, fat mass; FMI, fat mass index; HAZ, height-for-age z-score; HHS, household hunger score; MUAC, mid-upper arm circumference; RUTF, ready-to-use therapeutic food; SES, socioeconomic status; WAZ, weight-for-age z-score; WHZ, weight-for-height z-score. A dash (--) indicates zero observations. †:Data missing , n specified in brackets () or n/N | | | | |
