# Supplementary material for: Identifying risk and response to nutritional supplementation in severely underweight children missed by standard treatment criteria in Mali: A secondary prognostic analysis of data from a randomised controlled trial
Source: PLOS Glob Public Health. 2026 Nov 12;6(11):e0007525. doi: 10.1371/journal.pgph.0007525 (PMC13759120; doi:10.1371/journal.pgph.0007525)
Supplement: Supplementary file 4 [file pgph.0007525.s004.docx]

**S3 Table Outcome composition and reasons for referral or inpatient care among severely underweight children, by group**

|  | **Unsupplemented group (n=432)** | **Supplemented group (n=946)** |
| --- | --- | --- |
| Negative anthropometric outcome (composite) | 151/432 (35.0%) | 303/946 (32.0%) |
| No sustained WAZ gain (WAZ change < 0.15; supplemented definition) | — | 189/303 (62.4%) |
| Sustained WAZ decline (WAZ change < 0; unsupplemented definition) | 89/151 (58.9%) | — |
| Referred for severe wasting treatment | 66/151 (43.7%) | 111/303 (36.6%) |
| Low MUAC (< 125 mm) | 59/151 (39.1%) | 105/303 (34.7%) |
| Nutritional oedema | 15/151 (9.9%) | 24/303 (7.9%) |
| Referred for inpatient care | 11/151 (7.3%) | 23/303 (7.6%) |
| Symptoms requiring referral | 8/151 (5.3%) | 11/303 (3.6%) |
| Severe acute respiratory infection | 0/151 (0.0%) | 2/303 (0.7%) |
| Complicated malaria | 1/151 (0.7%) | 3/303 (1.0%) |
| Diarrhoea with dehydration | 0/151 (0.0%) | 1/303 (0.3%) |
| Fever > 39 C | 3/151 (2.0%) | 3/303 (1.0%) |
| Hypothermia < 35 C | 0/151 (0.0%) | 1/303 (0.3%) |
| Disease complication | 2/151 (1.3%) | 8/303 (2.6%) |
| Died | 0/151 (0.0%) | 3/303 (1.0%) |
| Any referral criterion met | 70/151 (46.4%) | 120/303 (39.6%) |
| *All values are shown as n/N (%), with the denominator given in each cell: the group total (432 unsupplemented; 946 supplemented) for the composite outcome, and the number of children with a negative anthropometric outcome (151 unsupplemented; 303 supplemented) for all component and referral rows. The anthropometric component is the absence of a sustained WAZ gain of ≥ 0.15 (i.e., WAZ change < 0.15) among supplemented children and a sustained WAZ change <0 among unsupplemented children. Values < 1% are reported where appropriate; 0 indicates no observed events. Referral rows count children with a negative anthropometric outcome who met each referral or inpatient-care criterion during follow-up; a child may meet more than one criterion, so the sub-categories do not sum to the total. These counts are not the same as the exit dispositions in S8 Table, which classifies each child by the single reason their follow-up ended (final disposition), whereas this table, restricted to children with a negative outcome, counts every referral or inpatient-care criterion met. Abbreviations: ARI, acute respiratory infection; MUAC, mid-upper arm circumference; WAZ, weight-for-age z score; °C, degrees Celsius; —, not applicable* | | |
