# Supplementary material for: Identifying risk and response to nutritional supplementation in severely underweight children missed by standard treatment criteria in Mali: A secondary prognostic analysis of data from a randomised controlled trial
Source: PLOS Glob Public Health. 2026 Nov 12;6(11):e0007525. doi: 10.1371/journal.pgph.0007525 (PMC13759120; doi:10.1371/journal.pgph.0007525)
Supplement: Supplementary file 5 [file pgph.0007525.s005.docx]

**S4 Table Full list of exposures associated with negative outcomes among severely underweight children not receiving nutritional support**

| **Exposure** | **Number at risk** | **Events** | **Crude RR (95% CI)** | **P-value** |
| --- | --- | --- | --- | --- |
| **Demographic** |  | | | |
| Age (months) | 432 | 151 | 0.99 (0.972, 0.999) | 0.043 |
| Age Group |  |  |  |  |
| Over 2 Years | 350 | 110 | 1.00 (Reference) |  |
| Under 2 Years | 82 | 41 | 1.59 (1.1, 2.26) | 0.011 |
| Sex |  |  |  |  |
| Female | 220 | 71 | 1.00 (Reference) |  |
| Male | 212 | 80 | 1.17 (0.85, 1.61) | 0.34 |
| **Anthropometry and Body Composition** |  | | | |
| Weight (kg) | 432 | 151 | 0.87 (0.77, 0.99) | 0.034 |
| Height (cm) | 432 | 151 | 0.97 (0.951, 0.997) | 0.031 |
| Weight-for-Height Z-score | 432 | 151 | 0.97 (0.78, 1.22) | 0.78 |
| Height-for-Age Z-score | 432 | 151 | 0.93 (0.77, 1.13) | 0.47 |
| Mid-Upper Arm Circumference (mm) | 432 | 151 | 0.97 (0.94, 1.01) | 0.17 |
| Fat-Free Mass (kg) | 168 | 60 | 0.83 (0.62, 1.1) | 0.22 |
| Fat-free mass index (FFMI) (kg/m^2^) | 155 | 55 | 1.02 (0.84, 1.2) | 0.86 |
| Fat Mass Index (FMI) (kg/m^2^) | 149 | 50 | 1.01 (0.83, 1.27) | 0.89 |
| Triceps Skinfold Z-score | 432 | 151 | 1.02 (0.84, 1.24) | 0.83 |
| Subscapular Skinfold Z-score | 432 | 151 | 0.99 (0.84, 1.17) | 0.88 |
| **Clinical** |  | | | |
| Hemoglobin Level (g/dl) | 427 | 147 | 1.08 (0.99, 1.17) | 0.089 |
| Anemia |  |  |  |  |
| No | 51 | 17 | 1.00 (Reference) |  |
| Yes | 376 | 130 | 1.04 (0.64, 1.78) | 0.89 |
| Recent Illness |  |  |  |  |
| No | 264 | 93 | 1.00 (Reference) |  |
| Yes | 168 | 58 | 0.98 (0.7, 1.36) | 0.90 |
| Malaria Result |  |  |  |  |
| Negative | 39 | 13 | 1.00 (Reference) |  |
| Positive | 121 | 37 | 0.92 (0.5, 1.79) | 0.79 |
| Vaccines Up to Date |  |  |  |  |
| No | 340 | 116 | 1.00 (Reference) |  |
| Yes | 92 | 35 | 1.12 (0.75, 1.61) | 0.57 |
| **Infant and Young Child Feeding** |  | | | |
| Currently Breastfeeding |  |  |  |  |
| No | 341 | 108 | 1.00 (Reference) |  |
| Yes | 91 | 43 | 1.49 (1.04, 2.11) | 0.026 |
| Breastfeeding Frequency |  |  |  |  |
| 1-4 Times | 25 | 13 |  |  |
| 5+ Times | 66 | 30 | 0.87 (0.47, 1.73) | 0.68 |
| Diet diversity score | 432 | 151 | 1.03 (0.87, 1.23) | 0.72 |
| Self Feeding |  |  |  |  |
| No | 25 | 14 | 1.00 (Reference) |  |
| Yes | 407 | 137 | 0.6 (0.36, 1.09) | 0.070 |
| **Caregiver and Household Characteristics** |  | | | |
| Caregiver Education Type |  |  |  |  |
| Informal | 339 | 122 | 1.00 (Reference) |  |
| Formal | 93 | 29 | 0.87 (0.57, 1.28) | 0.49 |
| Household Size Category |  |  |  |  |
| Large | 340 | 117 | 1.00 (Reference) |  |
| Small to Medium | 92 | 34 | 1.07 (0.72, 1.55) | 0.71 |
| Number of Children in Household | 432 | 151 | 0.99 (0.98, 1.002) | 0.20 |
| Number of Children Category | 432 | 151 | 0.98 (0.93, 1.03) | 0.49 |
| Large | 215 | 70 | 1.00 (Reference) |  |
| Small to Medium | 217 | 81 | 1.15 (0.83, 1.58) | 0.40 |
| Food Shortages (Past Month) |  |  |  |  |
| No | 394 | 138 | 1.00 (Reference) |  |
| Yes | 38 | 13 | 0.98 (0.53, 1.66) | 0.94 |
| Floor Material |  |  |  |  |
| Basic | 399 | 141 | 1.00 (Reference) |  |
| Cement | 33 | 10 | 0.86 (0.42, 1.54) | 0.64 |
| Socioeconomic Status (SES) Tertiles |  |  |  |  |
| Low | 154 | 58 | 1.00 (Reference) |  |
| Medium | 135 | 46 | 0.9 (0.61, 1.33) | 0.61 |
| High | 143 | 47 | 0.87 (0.59, 1.28) | 0.49 |
| Water Source |  |  |  |  |
| Improved Water Source | 265 | 90 | 1.00 (Reference) |  |
| Unimproved Water Source | 167 | 61 | 1.08 (0.77, 1.48) | 0.66 |
| Water Treatment |  |  |  |  |
| No | 326 | 116 | 1.00 (Reference) |  |
| Yes | 106 | 35 | 0.93 (0.63, 1.34) | 0.70 |
| Electricity Access |  |  |  |  |
| No | 324 | 119 | 1.00 (Reference) |  |
| Yes | 108 | 32 | 0.81 (0.54, 1.18) | 0.28 |
| Latrine Use |  |  |  |  |
| Latrine used | 199 | 67 | 1.00 (Reference) |  |
| No Latrine Used | 233 | 84 | 1.07 (0.78, 1.48) | 0.68 |
| Lean Season |  |  |  |  |
| No | 224 | 79 | 1.00 (Reference) |  |
| Yes | 208 | 72 | 0.98 (0.71, 1.35) | 0.91 |
| *Abbreviations: RR, risk ratio; CI, confidence interval; WHZ, weight-for-height z-score; HAZ, height-for-age z-score; MUAC, mid-upper arm circumference; FFMI, fat-free mass index; FMI, fat mass index; RDT, rapid diagnostic test; RUTF, ready-to-use therapeutic food; WAZ, weight-for-age z-score; SES, socioeconomic status; SD, standard deviation. Continuous exposures are modelled per unit increase. Reference categories are indicated as “Reference.” P values are derived from univariable regression models. Sample sizes vary across exposures due to missing data.* | | | | |
