# Supplementary material for: Identifying risk and response to nutritional supplementation in severely underweight children missed by standard treatment criteria in Mali: A secondary prognostic analysis of data from a randomised controlled trial
Source: PLOS Glob Public Health. 2026 Nov 12;6(11):e0007525. doi: 10.1371/journal.pgph.0007525 (PMC13759120; doi:10.1371/journal.pgph.0007525)
Supplement: Supplementary file 6 [file pgph.0007525.s006.docx]

**S5 Table Full list of exposures associated with positive anthropometric outcomes among children receiving nutritional support (unadjusted analysis)**

| **Exposure** | **Number at risk** | **Events** | **Crude RR (95% CI)** | **P** |
| --- | --- | --- | --- | --- |
| **Demographic** |  | | | |
| Age (months) | 946 | 643 | 1 (0.995, 1.008) | 0.64 |
| Age Group |  |  |  |  |
| Over 2 Years | 696 | 489 | 1.00 (Reference) |  |
| Under 2 Years | 250 | 154 | 0.88 (0.73, 1.05) | 0.15 |
| Sex |  |  |  |  |
| Female | 374 | 247 | 1.00 (Reference) |  |
| Male | 572 | 396 | 1.05 (0.9, 1.23) | 0.56 |
| **Anthropometry and Body Composition** |  | | | |
| Weight (kg) | 946 | 643 | 1.01 (0.95, 1.07) | 0.77 |
| Height (cm) | 946 | 643 | 1 (0.99, 1.01) | 0.59 |
| Weight-for-Height Z-score | 946 | 643 | 0.96 (0.86, 1.08) | 0.53 |
| Wasting Status |  |  |  |  |
| No | 848 | 570 | 1.00 (Reference) |  |
| Yes | 98 | 73 | 1.11 (0.86, 1.4) | 0.41 |
| Height-for-Age Z-score | 946 | 643 | 0.99 (0.9, 1.08) | 0.77 |
| Stunting Status |  |  |  |  |
| No | 33 | 21 | 1.00 (Reference) |  |
| Yes | 913 | 622 | 1.07 (0.71, 1.71) | 0.76 |
| Mid-Upper Arm Circumference | 946 | 643 | 1 (0.98, 1.01) | 0.64 |
| Fat-Free Mass (Absolute) | 346 | 248 | 1.1 (0.96, 1.25) | 0.17 |
| Fat Mass (Absolute) | 346 | 248 | 1.09 (0.95, 1.25) | 0.23 |
| Fat-free mass index (FFMI) | 294 | 212 | 0.96 (0.86, 1.05) | 0.37 |
| Fat Mass Index (FMI) | 288 | 208 | 1.05 (0.94, 1.19) | 0.40 |
| Triceps Skinfold Z-score | 946 | 643 | 0.98 (0.89, 1.07) | 0.65 |
| Subscapular Skinfold Z-score | 946 | 643 | 0.96 (0.89, 1.03) | 0.27 |
| **Clinical** |  | | | |
| Hemoglobin Level | 937 | 635 | 0.97 (0.93, 1.01) | 0.20 |
| Anemia |  |  |  |  |
| No | 113 | 72 | 1.00 (Reference) |  |
| Yes | 824 | 563 | 1.07 (0.85, 1.38) | 0.58 |
| Recent Illness |  |  |  |  |
| No | 539 | 334 | 1.00 (Reference) |  |
| Yes | 407 | 309 | 1.23 (1.05, 1.43) | 0.010 |
| Malaria Result |  |  |  |  |
| Negative | 83 | 50 | 1.00 (Reference) |  |
| Positive | 251 | 191 | 1.26 (0.93, 1.74) | 0.14 |
| Vaccines Up to Date |  |  |  |  |
| No | 726 | 497 | 1.00 (Reference) |  |
| Yes | 220 | 146 | 0.97 (0.8, 1.16) | 0.74 |
| **Infant and Young Child Feeding** |  | | | |
| Currently Breastfeeding |  |  |  |  |
| No | 710 | 504 | 1.00 (Reference) |  |
| Yes | 236 | 139 | 0.83 (0.685, 0.998) | 0.051 |
| Breastfeeding Frequency |  |  |  |  |
| 1-4 Times | 52 | 26 |  |  |
| 5+ Times | 184 | 113 | 1.23 (0.82, 1.92) | 0.34 |
| Diet diversity score | 946 | 643 | 1 (0.92, 1.09) | 0.96 |
| Self Feeding |  |  |  |  |
| No | 103 | 68 | 1.00 (Reference) |  |
| Yes | 843 | 575 | 1.03 (0.81, 1.34) | 0.80 |
| Went to Bed Hungry (Past Month) |  |  |  |  |
| No | 881 | 600 | 1.00 (Reference) |  |
| Yes | 65 | 43 | 0.97 (0.7, 1.31) | 0.85 |
| Going to Bed Hungry Frequency (Past Month) |  |  |  |  |
| Never(0 Times) | 881 | 600 | 1.00 (Reference) |  |
| Rarely (1-2 times) | 39 | 27 | 1.02 (0.67, 1.46) | 0.93 |
| Often (More than 2 times) | 26 | 16 | 0.9 (0.53, 1.43) | 0.69 |
| Treatment Type |  |  |  |  |
| 1 RUTF/day until WAZ ≥ -3 SD | 586 | 394 | 1.00 (Reference) |  |
| 1 RUTF/day until WHZ ≥ -2 SD | 243 | 169 | 1.03 (0.86, 1.24) | 0.71 |
| 125-215 kcal/kg/day RUTF | 117 | 80 | 1.02 (0.79, 1.29) | 0.89 |
| Treatment Arms |  |  |  |  |
| Standard arm | 360 | 249 | 1.00 (Reference) |  |
| Simplified arm | 586 | 394 | 0.97 (0.83, 1.14) | 0.73 |
| **Caregiver and Household Characteristics** |  | | | |
| Caregiver Education Type |  |  |  |  |
| Informal | 722 | 492 | 1.00 (Reference) |  |
| Formal | 224 | 151 | 0.99 (0.82, 1.18) | 0.91 |
| Caregiver Occupation |  |  |  |  |
| Formal | 25 | 15 | 1.00 (Reference) |  |
| Informal | 921 | 628 | 1.14 (0.71, 1.99) | 0.62 |
| Household Size Category |  |  |  |  |
| Large | 789 | 532 | 1.00 (Reference) |  |
| Small to Medium | 157 | 111 | 1.05 (0.85, 1.28) | 0.65 |
| Received food aid (Past month or Two Weeks ) |  |  |  |  |
| No | 892 | 605 | 1.00 (Reference) |  |
| Yes | 54 | 38 | 1.04 (0.74, 1.42) | 0.83 |
| Food Shortages (Past Month) |  |  |  |  |
| No | 854 | 577 | 1.00 (Reference) |  |
| Yes | 92 | 66 | 1.06 (0.82, 1.36) | 0.64 |
| Food Shortages Frequency (Past Month) |  |  |  |  |
| Never(0 Times) | 854 | 577 | 1.00 (Reference) |  |
| Rarely (1-2 times) | 54 | 39 | 1.07 (0.76, 1.46) | 0.69 |
| Often (More than 2 times) | 38 | 27 | 1.05 (0.7, 1.51) | 0.80 |
| Number of Children in Household | 946 | 643 | 1 (0.993, 1.003) | 0.43 |
| Number of Children Category | 946 | 643 | 1 (0.98, 1.02) | 0.93 |
| Large | 483 | 329 | 1.00 (Reference) |  |
| Small to Medium | 463 | 314 | 1 (0.85, 1.16) | 0.96 |
| Floor Material |  |  |  |  |
| Basic | 870 | 584 | 1.00 (Reference) |  |
| Cement | 76 | 59 | 1.16 (0.88, 1.5) | 0.29 |
| Socioeconomic Status (SES) Tertiles |  |  |  |  |
| Low | 306 | 211 | 1.00 (Reference) |  |
| Medium | 324 | 223 | 1 (0.83, 1.21) | 0.98 |
| High | 316 | 209 | 0.96 (0.79, 1.16) | 0.67 |
| Water Source |  |  |  |  |
| Improved Water Source | 629 | 434 | 1.00 (Reference) |  |
| Unimproved Water Source | 317 | 209 | 0.96 (0.81, 1.13) | 0.59 |
| Water Treatment |  |  |  |  |
| No | 720 | 489 | 1.00 (Reference) |  |
| Yes | 226 | 154 | 1 (0.83, 1.2) | 0.97 |
| Electricity Access |  |  |  |  |
| No | 666 | 457 | 1.00 (Reference) |  |
| Yes | 280 | 186 | 0.97 (0.81, 1.15) | 0.71 |
| Latrine Use |  |  |  |  |
| Latrine used | 528 | 354 | 1.00 (Reference) |  |
| No Latrine Used | 418 | 289 | 1.03 (0.88, 1.2) | 0.70 |
| Lean Season |  |  |  |  |
| No | 452 | 302 | 1.00 (Reference) |  |
| Yes | 494 | 341 | 1.03 (0.89, 1.21) | 0.68 |
| *Abbreviations: RR, risk ratio; CI, confidence interval; WHZ, weight-for-height z-score; HAZ, height-for-age z-score; MUAC, mid-upper arm circumference; FFMI, fat-free mass index; FMI, fat mass index; RDT, rapid diagnostic test; RUTF, ready-to-use therapeutic food; WAZ, weight-for-age z-score; SES, socioeconomic status; SD, standard deviation. Continuous exposures are modelled per unit increase. Reference categories are indicated as “Reference.” P values are derived from univariable regression models. Sample sizes vary across exposures due to missing data.* | | | | |
