# Supplementary material for: Identifying risk and response to nutritional supplementation in severely underweight children missed by standard treatment criteria in Mali: A secondary prognostic analysis of data from a randomised controlled trial
Source: PLOS Glob Public Health. 2026 Nov 12;6(11):e0007525. doi: 10.1371/journal.pgph.0007525 (PMC13759120; doi:10.1371/journal.pgph.0007525)
Supplement: Supplementary file 7 [file pgph.0007525.s007.docx]

**S6 Table : Sensitivity analyses: adjusted risk ratios (95% CI) for the principal baseline predictors under alternative outcome definitions and analytic approaches among unsupplemented children (negative anthropometric outcome)**

Each row is a sensitivity scenario; each cell is the adjusted RR (95% CI) for that predictor from a model refitted under the scenario. Panels correspond to the two exposure-defined groups and their group-specific composite outcome.

| **Sensitivity scenario** | **Events / N (%)** | **Age (per 1 month older)** | **Baseline weight (per 1 kg)** | **Baseline height (per 1 cm)** | **Currently breastfeeding (vs not)** |
| --- | --- | --- | --- | --- | --- |
| Primary analysis (negative outcome: ΔWAZ < 0) | 143 / 391 (36.6%) | 0.85 (0.71, 1.02) | 0.92 (0.62, 1.39) | 0.98 (0.93, 1.04) | 1.29 (0.79, 2.09) |
| Alternative threshold: ΔWAZ < −0.05 | 125 / 391 (32.0%) | 0.78 (0.64, 0.94) | 0.84 (0.55, 1.31) | 0.98 (0.92, 1.04) | 1.16 (0.70, 1.94) |
| Alternative threshold: ΔWAZ < −0.10 | 110 / 391 (28.1%) | 0.71 (0.58, 0.88) | 0.77 (0.50, 1.25) | 0.97 (0.91, 1.03) | 1.17 (0.68, 2.00) |
| Excluding children lost to follow-up | 132 / 351 (37.6%) | 0.88 (0.73, 1.05) | 0.89 (0.60, 1.38) | 0.98 (0.93, 1.04) | 1.35 (0.81, 2.24) |
| Excluding children who defaulted | 132 / 352 (37.5%) | 0.88 (0.73, 1.05) | 0.89 (0.59, 1.38) | 0.98 (0.93, 1.04) | 1.36 (0.82, 2.26) |
| Anthropometric-only outcome (morbidity/mortality events excluded) | 136 / 391 (34.8%) | 0.86 (0.72, 1.03) | 1.02 (0.68, 1.60) | 0.98 (0.93, 1.04) | 1.22 (0.74, 2.01) |
| AIC-selected model | 143 / 391 (36.6%) | 0.86 (0.72, 1.02) | 0.89 (0.78, 1.01) | 0.98 (0.95, 1.00) | 1.48 (0.96, 2.12) |
| *Abbreviations: AIC, Akaike information criterion; CI, confidence interval; RR, risk ratio; WAZ, weight-for-age z-score; ΔWAZ, change in weight-for-age z-score from baseline to week 12. Each cell presents the adjusted risk ratio (RR) with 95% confidence interval (CI) for the predictor, estimated using a Poisson generalised linear model with robust standard errors. Models were refitted under each stated sensitivity scenario and adjusted for the same covariates as the primary analysis (age, sex, season, and distance to the health facility). Only predictors that were statistically significant in the primary adjusted analysis are shown; all other candidate exposures remained non-significant across sensitivity scenarios and are omitted for brevity.* | | | | | |
