# Supplementary material for: Identifying risk and response to nutritional supplementation in severely underweight children missed by standard treatment criteria in Mali: A secondary prognostic analysis of data from a randomised controlled trial
Source: PLOS Glob Public Health. 2026 Nov 12;6(11):e0007525. doi: 10.1371/journal.pgph.0007525 (PMC13759120; doi:10.1371/journal.pgph.0007525)
Supplement: Supplementary file 8 [file pgph.0007525.s008.docx]

**S7 Table sensitivity analyses: adjusted risk ratios (95% CI) for the principal baseline predictors under alternative outcome definitions and analytic approaches among supplemented children ( positive anthropometric outcome)**

| **Sensitivity scenario** | **Events / N (%)** | **Recent illness (vs none)** |
| --- | --- | --- |
| Primary analysis (positive outcome: ΔWAZ ≥ 0.15) | 585 / 858 (68.2%) | 1.22 (1.03, 1.43) |
| Alternative threshold: ΔWAZ ≥ 0.10 | 620 / 858 (72.3%) | 1.18 (1.01, 1.38) |
| Alternative threshold: ΔWAZ ≥ 0.20 | 558 / 858 (65.0%) | 1.25 (1.06, 1.47) |
| Excluding children lost to follow-up | 570 / 821 (69.4%) | 1.22 (1.04, 1.44) |
| Anthropometric-only outcome (morbidity/mortality events excluded) | 596 / 858 (69.5%) | 1.19 (1.01, 1.39) |
| Excluding children recovered and discharged | 138 / 303 (45.5%) | 1.43 (1.02, 2.00) |
| AIC-selected model | 585 / 858 (68.2%) | 1.21 (1.03, 1.43) |
| *Abbreviations: AIC, Akaike information criterion; CI, confidence interval; RR, risk ratio; WAZ, weight-for-age z-score; ΔWAZ, change in weight-for-age z-score from baseline to week 12. Each cell presents the adjusted risk ratio (RR) with 95% confidence interval (CI) for the predictor, estimated using a Poisson generalised linear model with robust standard errors. Models were refitted under each stated sensitivity scenario and adjusted for the same covariates as the primary analysis (age, sex, season, and distance to the health facility). Only predictors that were statistically significant in the primary adjusted analysis are shown; all other candidate exposures remained non-significant across sensitivity scenarios and are omitted for brevity.* | | |
