# Supplementary material for: Identifying risk and response to nutritional supplementation in severely underweight children missed by standard treatment criteria in Mali: A secondary prognostic analysis of data from a randomised controlled trial
Source: PLOS Glob Public Health. 2026 Nov 12;6(11):e0007525. doi: 10.1371/journal.pgph.0007525 (PMC13759120; doi:10.1371/journal.pgph.0007525)
Supplement: Supplementary file 9 [file pgph.0007525.s009.docx]

.

**S8 Table. Missingness of candidate exposures by group**

Candidate exposures with any missing data, showing the number of children observed and the percentage missing within each group. All other candidate exposures listed in S1, S4 and S5 (demographic, remaining anthropometry, feeding, caregiver, and household/environmental variables) were complete (0% missing) in both groups

| **Exposure** | **Unsupplemented group (n = 432): n observed (% missing)** | **Supplemented group (n = 946): n observed (% missing)** |
| --- | --- | --- |
| Fat-free mass (kg) | 168 (61.1%) | 346 (63.4%) |
| Fat-free mass index (kg/m²) | 155 (64.1%) | 294 (68.9%) |
| Fat mass index (kg/m²) | 149 (65.5%) | 288 (69.6%) |
| Haemoglobin (g/dL) | 427 (1.2%) | 937 (1.0%) |
| Anaemia (Hb < 11 g/dL) | 427 (1.2%) | 937 (1.0%) |
| Malaria rapid diagnostic test result | 160 (63.0%) | 334 (64.7%) |
| *Abbreviations: Hb, haemoglobin. Numbers observed are the numbers at risk in S4 (unsupplemented) and S5 (supplemented) Tables; group totals are n = 432 (unsupplemented) and n = 946 (supplemented). Percentage missing = (group total − n observed) / group total. Body-composition measures (bioelectrical impedance; fat-free mass, fat mass and derived indices) and malaria rapid diagnostic testing were performed in a subset of children for operational reasons, accounting for their higher missingness; fat mass (absolute) was measured in the same subset as fat-free mass. Each regression model was fitted using complete-case data for the relevant exposure, so the number contributing to each model equals the number at risk in S4 and S5 Tables. Missingness cross-tabulated by outcome status within each group is shown in S7 Table* | | |
