# Supplementary material for: Identifying risk and response to nutritional supplementation in severely underweight children missed by standard treatment criteria in Mali: A secondary prognostic analysis of data from a randomised controlled trial
Source: PLOS Glob Public Health. 2026 Nov 12;6(11):e0007525. doi: 10.1371/journal.pgph.0007525 (PMC13759120; doi:10.1371/journal.pgph.0007525)
Supplement: Supplementary file 10 [file pgph.0007525.s010.docx]

**S9 Table: Missingness of candidate exposures by outcome status, within group**

| **Exposure** | **Unsupplemented group (n = 432)** | | **Supplemented group (n = 946)** | |
| --- | --- | --- | --- | --- |
|  | **Negative outcome (n = 151): n observed (% missing)** | **No negative outcome (n = 281): n observed (% missing)** | **Positive outcome (n = 643): n observed (% missing)** | **No positive outcome (n = 303): n observed (% missing)** |
| Fat-free mass (kg) | 60 (60.3%) | 108 (61.6%) | 248 (61.4%) | 98 (67.7%) |
| Fat-free mass index (kg/m²) | 55 (63.6%) | 100 (64.4%) | 212 (67.0%) | 82 (72.9%) |
| Fat mass index (kg/m²) | 50 (66.9%) | 99 (64.8%) | 208 (67.7%) | 80 (73.6%) |
| Haemoglobin (g/dL) | 147 (2.6%) | 280 (0.4%) | 635 (1.2%) | 302 (0.3%) |
| Anaemia (Hb < 11 g/dL) | 147 (2.6%) | 280 (0.4%) | 635 (1.2%) | 302 (0.3%) |
| Malaria rapid diagnostic test result | 50 (66.9%) | 110 (60.9%) | 241 (62.5%) | 93 (69.3%) |
| *Abbreviations: Hb, haemoglobin.For each exposure with missing data, the number of children with an observed value and the percentage missing within each outcome stratum, to assess whether missingness was associated with outcome status. Within the unsupplemented group children are split by the negative anthropometric outcome (151 with, 281 without); within the supplemented group by the positive anthropometric outcome (643 with, 303 without). Percentage missing = (stratum total − n observed) / stratum total. Hb, haemoglobin.* | | | | |
