# Supplementary material for: Identifying risk and response to nutritional supplementation in severely underweight children missed by standard treatment criteria in Mali: A secondary prognostic analysis of data from a randomised controlled trial
Source: PLOS Glob Public Health. 2026 Nov 12;6(11):e0007525. doi: 10.1371/journal.pgph.0007525 (PMC13759120; doi:10.1371/journal.pgph.0007525)
Supplement: Supplementary file 11 [file pgph.0007525.s011.docx]

**S10 Table: Follow-up cascade and outcome assignment by exposure group**

| **Follow-up status** | **Unsupplemented (n = 432)** | **Supplemented (n = 946)** |
| --- | --- | --- |
| Children included in the analysis | 432 (100.0%) | 946 (100.0%) |
| Completed 12 weeks of follow-up | 332 (76.9%) | 207 (21.9%) |
| Discontinued follow-up early, of whom: | 100 (23.1%) | 739 (78.1%) |
| Recovered and discharged | 0 (0.0%) | 619 (65.4%) |
| Referred for routine treatment (MUAC < 125 mm or oedema) | 52 (12.0%) | 72 (7.6%) |
| Required inpatient care (medical complications) | 0 (0.0%) | 0 (0.0%) |
| Died | 0 (0.0%) | 5 (0.5%) |
| Defaulted | 47 (10.9%) | 40 (4.2%) |
| Lost to follow-up (no exit reason recorded) | 1 (0.2%) | 3 (0.3%) |
| Outcome assigned from last available measurement (LOCF) | 100 (23.1%) | 739 (78.1%) |
| *Values are n (%) of the group total. Follow-up was discontinued early for recovery, referral for routine treatment on developing MUAC < 125 mm or nutritional oedema, inpatient care for medical complications, death, or default. Outcome status was assigned from each child's last available anthropometric measurement (last observation carried forward); children referred for routine treatment, hospitalised for medical complications, or who died were classified as having a negative anthropometric outcome irrespective of their weight-for-age trajectory, as these events are components of the composite negative outcome.* | | |
