# Supplementary material for: Identifying risk and response to nutritional supplementation in severely underweight children missed by standard treatment criteria in Mali: A secondary prognostic analysis of data from a randomised controlled trial
Source: PLOS Glob Public Health. 2026 Nov 12;6(11):e0007525. doi: 10.1371/journal.pgph.0007525 (PMC13759120; doi:10.1371/journal.pgph.0007525)
Supplement: Supplementary file 12 [file pgph.0007525.s012.docx]

**S11 Table Age distribution of currently breastfed children with a negative anthropometric outcome (unsupplemented group)**

Among currently breastfed children in the unsupplemented group, the distribution of age at enrolment and of the negative anthropometric outcome across age bands.

| **Age band at enrolment (months)** | **All breastfed children n (%)** | **Breastfed children with a negative anthropometric outcome n (col %)** | **Risk within age band n/N (%)** |
| --- | --- | --- | --- |
| 6–11 | 5 (5.5%) | 3 (7.0%) | 3/5 (60.0%) |
| 12–23 | 63 (69.2%) | 32 (74.4%) | 32/63 (50.8%) |
| 24–35 | 23 (25.3%) | 8 (18.6%) | 8/23 (34.8%) |
| 36–59 | 0 (0.0%) | 0 (0.0%) | 0/0 (—) |
| **Total** | 91 (100.0%) | 43 (100.0%) | 43/91 (47.3%) |
| *Restricted to children in the unsupplemented group who were currently breastfeeding at enrolment (n = 91). Column %: the percentage of all breastfed children with a negative anthropometric outcome who fell in that age band (column sums to 100%). Risk within age band: the number with a negative anthropometric outcome divided by the number of breastfed children in that age band. The negative anthropometric outcome is defined as in the main text (sustained WAZ change < 0 at any point over 0–12 weeks, death, hospitalisation, or development of MUAC < 125 mm).*  *Exclusive breastfeeding duration and age at introduction of complementary foods were not recorded in the parent trial; only current breastfeeding status at enrolment was available. This table therefore describes the age distribution of breastfed children with a negative outcome but cannot test whether these outcomes cluster around the weaning transition (the “first pathogenic exposure” hypothesis). WAZ, weight-for-age Z-score; MUAC, mid-upper arm circumference.* | | | |
